# Association of physical activity with risk of hepatobiliary diseases in China: a prospective cohort study of 0.5 million people

**DOI:** 10.1136/bjsports-2020-102174

**Published:** 2020-08-21

**Authors:** Yuanjie Pang, Jun Lv, Christiana Kartsonaki, Canqing Yu, Yu Guo, Huaidong Du, Derrick Bennett, Zheng Bian, Yiping Chen, Ling Yang, Iain Turnbull, Hao Wang, Hui Li, Michael V Holmes, Junshi Chen, Zhengming Chen, Liming Li

**Affiliations:** 1 Department of Epidemiology and Biostatistics, School of Public Health, Peking University Health Science Center, Beijing, China; 2 Clinical Trial Service Unit & Epidemiological Studies Unit (CTSU), Nuffield Department of Population Health, Big Data Institute Building, Roosevelt Drive, University of Oxford, Oxford, UK; 3 Medical Research Council Population Health Research Unit (MRC PHRU), Nuffield Department of Population Health, University of Oxford, Oxford, UK; 4 Chinese Academy of Medical Sciences, Beijing, China; 5 National Institute for Health Research Oxford Biomedical Research Centre, Oxford University Hospital, Oxford, UK; 6 Zhejiang Center for Disease Prevention and Control, Hangzhou, China; 7 Liuzhou Chinese Medicine Hospital, Liuzhou, China; 8 National Center for Food Safety Risk Assessment, Beijing, China

**Keywords:** epidemiology, physical activity, liver, cancer

## Abstract

**Objective:**

There is limited prospective evidence on the association of physical activity with hepatobiliary cancer subtypes and other major hepatobiliary diseases, especially in China. We aimed to quantify the associations with risk of these diseases.

**Methods:**

The study population involved 460 937 participants of the prospective China Kadoorie Biobank aged 30–79 years from 10 diverse areas in China without history of cancer or hepatobiliary disease at baseline. Cox regression was used to estimate adjusted hazard ratios (HRs) for each disease associated with self-reported total and domain-specific physical activity (occupational and non-occupational, ie, leisure time, household and commuting).

**Results:**

During ~10 years of follow-up, 22 012 incident cases of hepatobiliary diseases were recorded. The overall mean (SD) total physical activity was 21.2 (13.9) metabolic equivalent of task (MET)-hours/day, with 62% from occupational activity. Total physical activity was inversely associated with hospitalised non-alcoholic fatty liver disease (HR comparing top vs bottom quintile: 0.62, 95% confidence interval (CI) 0.53 to 0.72), viral hepatitis (0.73, 95% CI 0.62 to 0.87), cirrhosis (0.76, 95% CI 0.66 to 0.88) and liver cancer (0.81, 95% CI 0.71 to 0.93), as well as gallstone disease (0.86, 95% CI 0.81 to 0.90), gallbladder cancer (0.51, 95% CI 0.32 to 0.80) and biliary tract cancer (0.55, 95% CI 0.38 to 0.78). The associations for occupational physical activity were similar to those for total physical activity, but for non-occupational physical activity they differed by disease subtype. For leisure-time physical activity, there was an inverse association with liver cancer and an inverse trend for gallstone disease (HR comparing ≥7.5 MET-hours/day with none: 0.83, 95% CI 0.75 to 0.91 and 0.82, 95% CI 0.66 to 1.01).

**Conclusion:**

Among Chinese adults, high total physical activity, particularly occupational physical activity, was inversely associated with risk of major hepatobiliary cancers and diseases, including non-alcoholic fatty liver disease, cirrhosis and certain types of cancer.

## Introduction

Hepatobiliary disease is a major cause of mortality and morbidity and accounted for 2.4 million deaths globally in 2017.^1^ Worldwide, about half of deaths from liver cancer and 15% from cirrhosis occur in China.^1^ In recent decades, the age-standardised mortality rates from liver cancer and cirrhosis have been declining in China due to elimination of aflatoxin and control of hepatitis B virus (HBV),^2 3^ whereas the incidence rate of non-alcoholic fatty liver disease has been rising due to increasing prevalence of a sedentary lifestyle and Westernised diets.^3^ The most common type of gallbladder disease is gallstones, affecting 10%–20% of the global adult population.^4 5^ East Asian countries, including China, have been traditionally considered as areas with low risk of gallstones.^4^ Gallbladder and biliary tract cancer (GBTC) is a rare cancer, and its incidence mirrors that of gallstones, suggesting a similar risk factor profile for both diseases.^1^ Gallbladder disease and cancer may increase in China due to increasing prevalence of lifestyle-related risk factors, particularly obesity and physical inactivity.

Possible lifestyle-related risk factors for hepatobiliary diseases include smoking, alcohol and dietary factors, as well as metabolic risk factors including general and central adiposity, low leisure-time physical activity and diabetes.^6–10^ For physical activity, prospective studies have shown inverse associations of total and leisure-time physical activity with liver cancer, non-alcoholic fatty liver disease and gallbladder disease,^11–15^ but there is limited evidence on other hepatobiliary cancers and diseases. Hepatobiliary diseases, including chronic liver disease and gallbladder disease, lie on the pathophysiological pathway to hepatobiliary cancers and may share metabolic risk factors related to the insulin resistance syndrome.^5–8 16^ As insulin resistance has been hypothesised as a possible mechanism underlying the association of physical activity with hepatobiliary cancer,^17^ assessing the associations of physical activity with non-cancer outcomes may provide valuable insights into disease aetiology and inform prevention. Moreover, the majority of previous studies were in Western populations, where the patterns of metabolic risk factors, physical activity and diseases differ from those in China. For example, HBV accounts for 45% of liver cancer and cirrhosis in China,^2^ whereas alcohol accounts for 30% of liver cancer^18^ and 30%–40% of cirrhosis in Western countries.^19 20^ Occupational physical activity is the largest domain in China,^21^ whereas leisure-time physical activity is the largest domain in Western populations.^22^ However, there is limited evidence on domain-specific physical activity and hepatobiliary diseases both in China and in Western countries. Reliable assessment of the associations of physical activity with many different hepatobiliary diseases in Chinese may help inform disease prevention and better understanding of aetiology.

Our aims were to (1) quantify the associations of total, occupational and non-occupational physical activity with the risk of hepatobiliary diseases; (2) examine whether these associations differ by region, sex, age, sedentary leisure time, work activity intensity and different levels of baseline risk factors; and (3) assess whether additional adjustment for metabolic risk factors (eg, adiposity and diabetes) could alter the associations of physical activity with hepatobiliary diseases. We hypothesised that (1) total and occupational physical activity was associated with lower risk of hepatobiliary diseases; and (2) the associations of non-occupational physical activity with hepatobiliary diseases differed by domain of physical activity due to the different nature of activity in Chinese compared with Western populations (eg, unique patterns of leisure-time physical activity, urban–rural differences).

## Methods

### Study population

The study recruited 512 715 participants aged 30–79 years from 10 geographically defined localities (five urban and five rural) in China during 2004–2008.^23^ The study areas were selected to provide diversity in risk exposure and disease patterns, while taking into account population stability, quality of mortality and morbidity registries, capacity, and long-term commitment within the areas. At local study assessment clinics, participants completed an interviewer-administered, laptop-based questionnaire on sociodemographic characteristics, lifestyle factors, personal and family medical history, and current medication. A range of physical measurements were recorded by trained technicians, including height, weight, hip and waist circumference, bioimpedance, lung function, blood pressure, and heart rate, using calibrated instruments with standard protocols. All participants provided a 10 mL non-fasting blood sample for immediate on-site test of random plasma glucose (SureStep Plus metre; LifeScan, Johnson & Johnson) and HBsAg (ACON Biotech).

### Assessment of physical activity

The questions on physical activity and sedentary leisure time were adapted from validated questionnaires used in several other studies, including the European Prospective Investigation into Cancer and Nutrition (EPIC) and the Shanghai Women’s Health Study,^24 25^ with some additional modifications after a China Kadoorie Biobank (CKB) pilot study. At baseline and subsequent resurveys, participants were asked about the frequency, duration and type (intensity) of physical activity in four domains (ie, occupation, commuting, housework and leisure-time exercise) during the past 12 months. To quantify the amount of physical activity, metabolic equivalent of tasks (MET) from the 2011 update of a compendium of physical activities was used.^26^ The MET value for a particular type of physical activity represents the ratio of the energy expended per kilogram of body weight per hour during that activity relative to that expended when sitting quietly. The number of hours spent per day participating in each activity was multiplied by the MET value for that activity, and the daily amount of total physical activity was obtained by summing the MET-hours/day for activities related to occupational and non-occupational (ie, commuting, housework and non-sedentary leisure time) activities. Hours spent per day on sedentary leisure-time activities (such as television watching, reading, and playing cards or mahjong) and sleeping were also recorded, but were not included in the physical activity calculation. Online supplementary table 1 shows the types, MET values, codes and intensity categories of physical activity.

10.1136/bjsports-2020-102174.supp1 Supplementary data

### Follow-up for morbidity and mortality

The vital status of each participant was determined periodically through the China Center for Disease Control and Prevention’s Disease Surveillance Points system and national health insurance system,^27^ supplemented by regular checks against local residential and administrative records and by annual active confirmation through street committees or village administrators. Additional information about major diseases and any episodes of hospitalisation was collected through linkages, via each participant’s unique national identification number, with disease registries (for cancer, ischaemic heart disease, stroke and diabetes) and national health insurance claims databases, which have almost universal coverage in the study areas. All events were coded using the International Classification of Diseases, 10th Revision (ICD-10) by trained staff who were blinded to baseline information and reviewed centrally for consistency.^23^ The classification and distribution of hepatobiliary diseases are shown in online supplementary table 2. By 1 January 2017 (censoring date for the present analyses), 44 066 (8.6%) participants had died and 4751 (1.0%) were lost to follow-up.

### Statistical analysis

We excluded individuals with history of cancer (n=2578), cirrhosis or hepatitis (n=6139), a positive HBsAg test (n=17 170), or gallbladder disease at baseline (n=25 891), leaving 460 937 individuals for the main analysis. Mean values and prevalence of baseline characteristics were calculated for categories of total physical activity at baseline, standardised to age (in 5-year groups), sex and area structure of the CKB population.

Cox proportional hazards models with age as the underlying time scale and delayed entry at age at baseline were used to estimate HRs of specific disease incidence associated with physical activity levels. Models were stratified by sex and study area, and adjusted for age at baseline, education, household income, smoking, alcohol, self-rated health, diabetes, cardiovascular disease, respiratory disease, rheumatoid arthritis and sedentary leisure time. Physical activity was categorised by splitting at quintiles in order to assess the shape of the association. If the association was linear, then physical activity was also modelled as a continuous variable to estimate the risk associated with a 4 MET-hours/day higher level of physical activity. Separate analyses were conducted for occupational and non-occupational physical activity with mutual adjustment. For exposure variables with more than two categories, all HRs are presented with ‘floating’ standard errors (SEs) to facilitate comparisons between groups.^28^ The CKB estimates for physical activity and hepatobiliary cancers were meta-analysed with estimates from published prospective cohort studies using a random-effects meta-analysis. Details of the study selection are reported in online supplementary figure 1. Regression dilution was corrected for using the McMahon-Peto method (online supplementary table 3).^29–32^ Further details of the statistical analysis including covariates, regression dilution and sensitivity analyses are reported in online supplementary eMethods. Statistical analysis was done using R 2.14.2.

## Results

### Baseline characteristics of participants by physical activity

Among all 460 937 participants included, the mean (SD) age at baseline was 52 (10.5) years. The mean (SD) total physical activity was 21.2 (13.9) MET-hours/day. Occupational physical activity accounted for a higher proportion of total physical activity in men than in women (72% vs 57%). The patterns of non-occupational physical activity differed by sex and urbanicity (online supplementary table 4). For example, women had almost threefold higher levels of household activity than men, while participants in urban areas had almost tenfold higher levels of leisure-time activity than those in rural areas (mean 1.7 vs 0.2 MET-hours/day). Participants with higher total physical activity were more likely to be men, younger, from rural areas and had lower levels of sedentary leisure time (table 1). They were more likely to have lower levels of systolic blood pressure, random plasma glucose and adiposity, and less likely to have diabetes, cardiovascular disease and hypertension.

### Associations of total physical activity with hepatobiliary diseases

**Figure 1:**
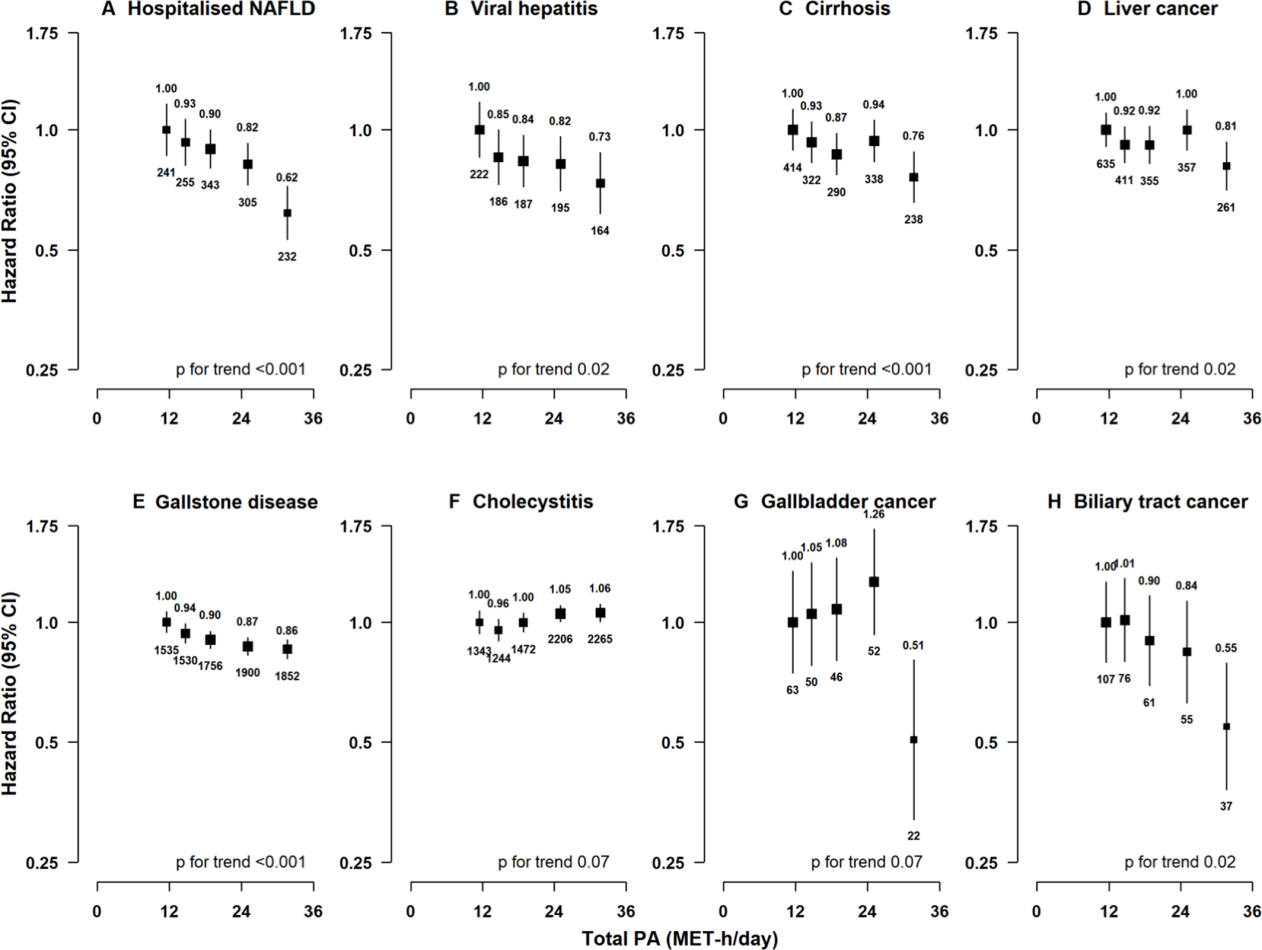
Associations of total physical activity with risk of hepatobiliary diseases. Models were stratified by sex and region, and adjusted for age at baseline, education, household income, smoking, alcohol, self-rated health, diabetes, cardiovascular disease, respiratory disease, rheumatoid arthritis and sedentary leisure time. Time since birth was used as the underlying time scale with delayed entry at age at baseline. HRs are plotted against the mean level in each category of physical activity. Log scale is used for the y axis. The squares represent HR and the vertical lines represent 95% CI. The area of the squares is inversely proportional to the variance of the log HR. The numbers above the vertical lines are point estimates for HR, and the numbers below the lines are the number of events. MET, metabolic equivalent of task; NAFLD, non-alcoholic fatty liver disease; PA, physical activity.

For liver diseases, total physical activity showed inverse associations with hospitalised non-alcoholic fatty liver disease, viral hepatitis, cirrhosis and liver cancer (figure 1 and online supplementary table 5), similar across these subtypes (p for heterogeneity 0.13). There was no evidence of an association with alcoholic liver disease (HR 0.92 (95% CI 0.70–1.22)), possibly due to the small number of cases involved. For gallbladder diseases, total physical activity showed inverse associations with gallstone disease, gallbladder cancer and biliary cancer but not with cholecystitis (figure 1 and online supplementary table 5), which were similar for GBTC (p for heterogeneity 0.34) but differed for gallstone disease and cholecystitis (p for heterogeneity 0.02). Likelihood ratio tests showed evidence of significant non-linear associations for gallstone disease and biliary tract cancer (p for non-linearity=0.01 and 0.02) and no strong evidence of non-linear associations for other diseases (p for non-linearity=0.06–0.77; online supplementary figure 2). Similar associations were observed for intrahepatic bile duct cancer and liver cancer and for extrahepatic bile duct cancer and biliary tract cancer (online supplementary table 6).

### Associations of domain-specific physical activity with hepatobiliary diseases

**Figure 2:**
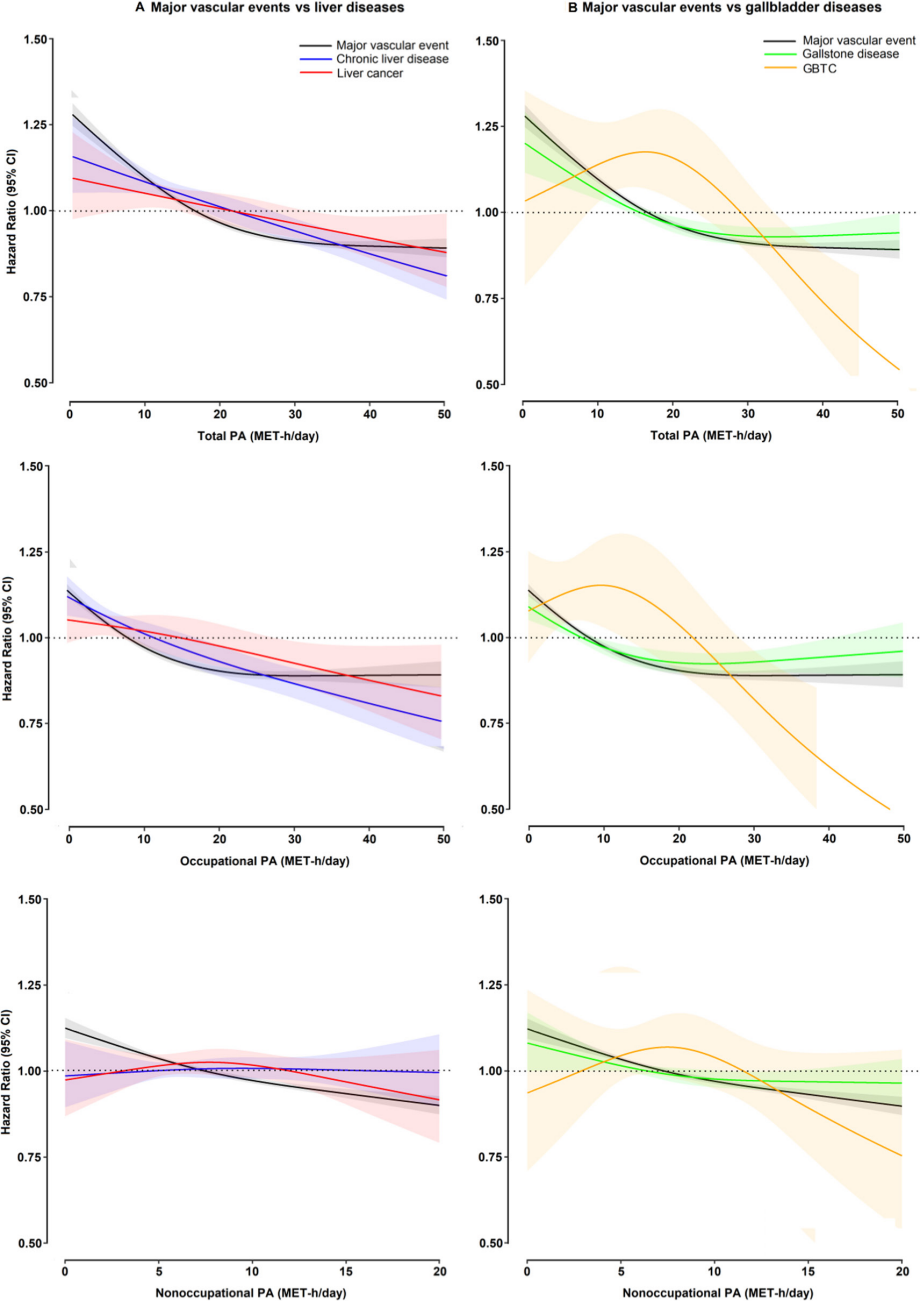
Associations of physical activity with risk of major coronary events and hepatobiliary diseases. Panel A denotes liver diseases and panel B denotes gallbladder diseases. Restricted cubic splines were calculated using three fixed knots at 10%, 50% and 90% quintiles. figure 1Major coronary events denote major adverse cardiovascular events (ICD-10: I21-I23 from any cause; I20, I24 or I25 only when fatal). Participants with history of cardiovascular diseases were excluded from the analysis of physical activity and major coronary events. GBTC, gallbladder and biliary tract cancer; ICD, International Classification of Diseases; MET, metabolic equivalent of task; PA, physical activity.

For domain-specific physical activity, the shape and strength of the inverse associations with specific disease type were similar for occupational and total physical activity, whereas there were no clear associations for non-occupational physical activity (online supplementary figures 3–4). When compared with major coronary events (major coronary events; ICD-10: I21-I23 and I20, I24 or I25 fatal only), the threshold above which an inverse association was observed for both total and occupational physical activity was higher for chronic liver disease, liver cancer and GBTC and similar for gallstone disease (figure 2). For non-occupational physical activity, there were inverse associations with major coronary events and no clear patterns for hepatobiliary cancers and diseases.

**Figure 3:**
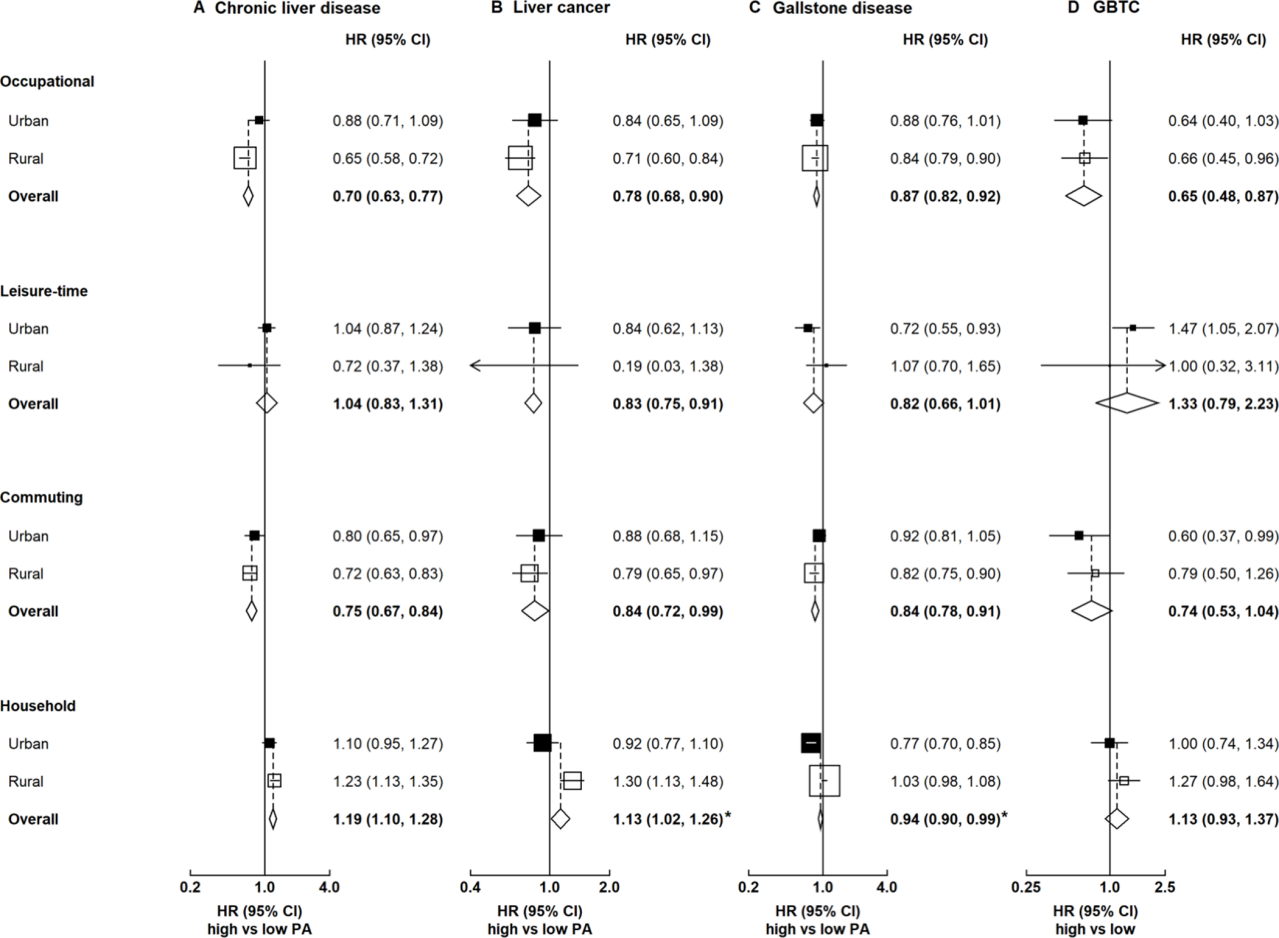
Associations of domain-specific physical activity with hepatobiliary diseases. Boxes represent the HR associated with hepatobiliary disease comparing high and low categories of physical activity, with the size of the box inversely proportional to the variance of the log HR. Diamonds represent summary HR in both urban and rural areas. Asterisk (*) denotes heterogeneity by urbanicity (p value for heterogeneity <0.05). The amount of occupational and non-occupational physical activity was categorised by splitting at quintiles, while the amount of commuting and household physical activity was categorised by splitting at tertiles. In the analysis by urbanicity, region-specific cut-off points were used. For leisure-time physical activity, the HR was comparing ≥7.5 MET-hours/day (recommended by the 2010 WHO guidelines^62^) with none. GBTC, gallbladder and biliary tract cancer; MET, metabolic equivalent of task; PA, physical activity.

As the patterns of physical activity differed in urban and rural areas, the associations of domain-specific physical activity with hepatobiliary diseases were presented in urban and rural areas separately (figure 3). We combined chronic liver disease and GBTC in subsequent domain-specific analyses because the associations of physical activity with hepatobiliary diseases did not differ across disease subtypes. Overall the associations with hepatobiliary diseases did not differ between occupational and non-occupational physical activity, except for chronic liver disease and liver cancer in rural areas (p value for heterogeneity <0.001; figure 3 and online supplementary figure 5). For chronic liver disease and liver cancer, there were inverse associations for commuting physical activity, while an inverse association for leisure-time physical activity was only observed for liver cancer. In contrast, there were positive associations for household physical activity in rural areas and no clear associations in urban areas. For gallstone disease, inverse trends were observed across four domains. For GBTC, there were inverse trends for occupational and commuting physical activity but no clear associations for leisure-time and household physical activity, probably because of the small number of events. The associations of domain-specific physical activity with hepatobiliary diseases did not differ between urban and rural areas, except for household physical activity with liver cancer and gallstone disease (online supplementary figure 5).

### Subgroup and sensitivity analyses

Overall the associations of physical activity with hepatobiliary disease did not differ by sex except for occupational physical activity and GBTC (p value for heterogeneity 0.03; online supplementary figure 6). For other population subgroups, the associations of physical activity with hepatobiliary diseases were consistent across population subgroups, with the exception of chronic liver disease by education and smoking (online supplementary figures 7–8). Of note, the associations of physical activity with hepatobiliary diseases did not differ by sedentary leisure time and work activity level. The associations for physical activity with hepatobiliary diseases and cancers were largely unaltered when excluding the first 5 years of follow-up and participants with chronic diseases or poor self-rated health at baseline (online supplementary table 7 and online supplementary figure 9). To explore potential bias, we selected accidental death (ICD-10: V01-X59, excluding cycling accidents (V10-19) and intentional self-harm (X60-84)) as a negative control outcome, which had a similar confounding structure as observed with liver cancer (online supplementary table 8). As shown in online supplementary table 9 and online supplementary figure 10, there were no associations for total, occupational and non-occupational physical activity.

**Figure 4:**
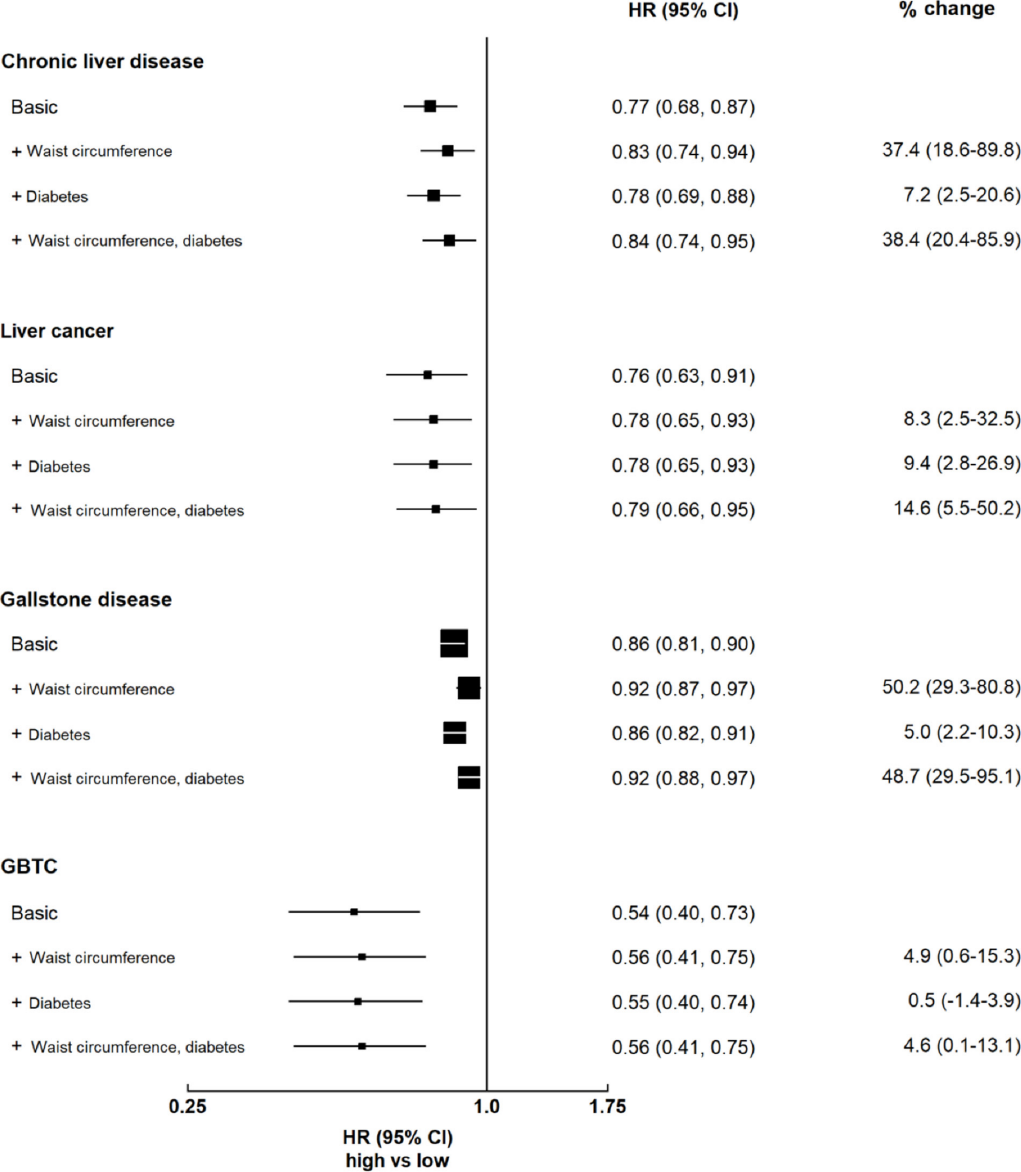
Per cent change in the association of total physical activity with hepatobiliary diseases with additional adjustment for adiposity and diabetes. Boxes represent the HR associated with hepatobiliary cancer comparing high versus low categories of physical activity, with the size of the box inversely proportional to the variance of the log HR. Models were stratified by sex and region, and adjusted for age at baseline, education, household income, smoking, alcohol, self-rated health, diabetes, cardiovascular disease, respiratory disease, rheumatoid arthritis and sedentary leisure time. For chronic liver disease and liver cancer, the first 5 years of follow-up were excluded. GBTC, gallbladder and biliary tract cancer.

For chronic liver disease, the inverse association for total physical activity attenuated by 37.4% with additional adjustment for waist circumference (95% CI 18.6% to 89.8%), while additional adjustment for diabetes changed little of the inverse association (7.2%, 95% CI 2.5% to 20.6%; figure 4). The attenuation was greater in men than in women (waist circumference: 47.1% and 21.5%; diabetes: 8.2% and 2.2%; online supplementary table 10). For gallbladder disease, the inverse association for total physical activity attenuated by 50.2% with additional adjustment for waist circumference (50.2% (29.3%–80.8%)), and there was little change when additionally adjusting for diabetes (5.0% (2.2%–10.3%)). The decrease was similar by sex (online supplementary table 10). In contrast, additional adjustment for waist circumference or diabetes altered little of the inverse associations of total physical activity with liver cancer or GBTC (figure 4).

### Meta-analyses of CKB with previous prospective studies on hepatobiliary cancers

**Figure 5:**
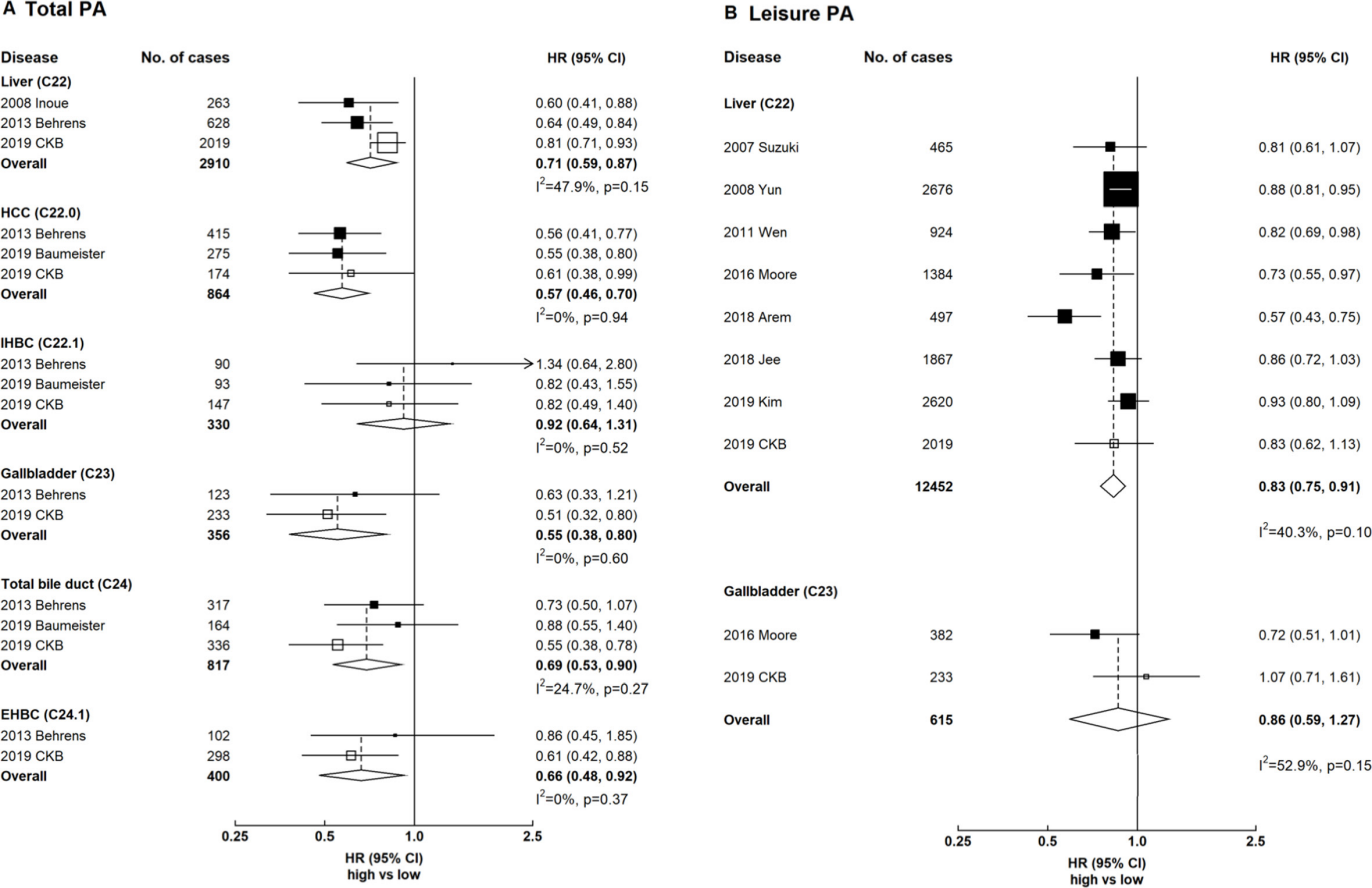
Meta-analysis of prospective studies on physical activity and hepatobiliary cancers. Panel A denotes total physical activity and panel B denotes leisure physical activity. Boxes represent the HR associated with hepatobiliary cancer comparing high versus low categories of physical activity, with the size of the box inversely proportional to the variance of the log HR. Open boxes represent previously published studies, and the black boxes represent CKB estimates. Diamonds represent summary HR for overall. Within categories HRs are ordered according to their year of publication. Estimates and 95% CI of the summary HR are in bold. References for previous studies: Suzuki 2007, PMID 18260704; Inoue 2008, PMID 18599492; Behrens 2013, PMID 23354983; Baumeister 2019, PMID 30582978; Yun 2008, PMID 19077256; Wen 2011, PMID 21846575; Moore 2016, PMID 27183032; Arem 2018, PMID 29533015; Jee 2018, PMID 29914427; Kim 2019, PMID 30872551. CKB, China Kadoorie Biobank; EHBC, extrahepatic bile duct cancer; HCC, hepatocellular carcinoma; IHBC, intrahepatic bile duct cancer; PA, physical activity.

Total physical activity showed inverse associations with liver, gallbladder and extrahepatic biliary tract cancers (figure 5). The HRs comparing the top and bottom categories of total physical activity were 0.71 (95% CI 0.59 to 0.87) for liver cancer, 0.55 (95% CI 0.38 to 0.80) for gallbladder cancer and 0.66 (95% CI 0.48 to 0.92) for extrahepatic bile duct cancer, with little between-study heterogeneity (I^2^=47.9%, I^2^=0% and I^2^=0%, respectively). For liver cancer subtypes, there was an inverse association for hepatocellular carcinoma and no association for intrahepatic bile duct cancer (0.57 (95% CI 0.46 to 0.70) and 0.92 (95% CI 0.64 to 1.31)), with little between-study heterogeneity (I^2^=0). For leisure-time physical activity, there was an inverse association for liver cancer (0.83, 95% CI 0.75 to 0.91) and no association for gallbladder cancer (0.86, 95% CI 0.59 to 1.27), with moderate between-study heterogeneity (I^2^=40.3% and I^2^=52.9%).

## Discussion

In this Chinese population, total physical activity was inversely associated with several major subtypes of hepatobiliary diseases. The associations for occupational physical activity mirrored those with total physical activity. For non-occupational physical activity, the associations with hepatobiliary diseases differed by domain, possibly reflecting residual confounding. The inverse associations of total physical activity attenuated by ~40% for chronic liver disease and ~50% for gallstone disease when we also adjusted for central adiposity. Our main results remained largely unchanged when we excluded (1) the first 5 years of follow-up and (2) participants with chronic diseases or poor self-rated health at study baseline.

### Extending the previous findings in Western populations

Prospective cohort studies in Western populations reported an inverse association for hepatocellular carcinoma but no associations for other subtypes, including intrahepatic bile duct cancer, extrahepatic bile duct cancer and gallbladder cancer.^11 12^ However, these studies involved fewer than 800 hepatobiliary cancer cases and may have limited statistical power. In this CKB population, there were inverse associations between total physical activity and liver cancer and GBTC.

When meta-analysed with previous cohort studies, there were inverse associations for gallbladder cancer and extrahepatic bile duct cancer. For leisure-time physical activity, the inverse association in CKB is consistent with previous studies in Western and East Asian populations,^13 33–37^ including a cohort study of 416 175 participants and 924 liver cancer cases in Taiwan (HR comparing ≥7.5 MET-hours/day vs none: 0.83 (0.75–0.91) in CKB vs 0.82 (0.69–0.98) in that study).^34^ For gallbladder cancer, a pooled analysis of 12 cohort studies in the USA and Europe with 382 cases reported an inverse association.^13^ However, no association was observed in CKB probably because of the narrower range of leisure-time physical activity (median 0.1 MET-hours/day in CKB vs 8–11 MET-hours/day in the pooled analysis).^13^

For non-alcoholic fatty liver disease, a meta-analysis of six cohort studies with 32 657 cases showed a 21% lower risk comparing high and low categories of physical activity, with similar associations for total and leisure-time physical activities.^14^ In CKB, the finding for total physical activity was consistent with previous studies, whereas there was no clear association for leisure-time physical activity (online supplementary figure 11). A previous randomised controlled trial in Chinese people showed that moderate to vigorous exercise was associated with lower risk of non-alcoholic fatty liver disease.^38^ Therefore, the null association might be due to the low intensity of leisure-time physical activity in CKB. For gallbladder diseases, a meta-analysis of eight cohort studies and 6197 cases reported inverse associations for total and leisure-time physical activities,^15^ consistent with findings in CKB (online supplementary figure 11). In CKB, we found an inverse association for gallstone disease and no association for cholecystitis (p for heterogeneity 0.02), consistent with previous studies showing stronger associations of other metabolic risk factors with gallstone disease than cholecystitis.^9 39^

### Associations in rural and urban regions

In our CKB population, we report heterogeneity in the associations of domain-specific physical activity with chronic liver disease and liver cancer in rural areas. The positive associations of household physical activity in rural areas might reflect confounding by infections.

HBV is a major risk factor for liver cancer and chronic liver disease,^3^ and household contact (contaminated blood or body fluids) is a major mode of transmission.^40^ In CKB, household physical activity was directly calculated from household hours (no information on types collected) and might be an indicator of household contact. Analysis of the China Nutritional Health Survey showed that the proportion of household physical activity involving direct household contact was much higher in rural than in urban areas (66.9% vs 31.9%).^41^ In contrast, no associations were observed for household physical activity and gallstone disease and GBTC, which were unlikely to be associated with infectious agents.^5^ In addition, when accidental death was used as a negative control outcome, there were no overall associations of total, occupational and non-occupational physical activity with risk of accidental death (online supplementary table 9 and online supplementary figure 10). This suggests that potential confounding and bias from other sources are likely to be minimal.

### On causality

Although the current study findings cannot be used to establish causality, the inverse associations of physical activity with hepatobiliary diseases may not be fully explained by residual confounding and reverse causality. Early prospective studies conducted in Western populations reported inverse associations of occupational physical activity with major chronic diseases, whereas more recent studies have shown inconsistent associations.^42 43^ This change over time might reflect confounding by socioeconomic status. In CKB, we adjusted for education and household income, which have been shown to be good indicators of socioeconomic status in this Chinese population.^44^ We further showed that the inverse associations were consistent in different birth cohorts in CKB (online supplementary table 11). In addition, the EPIC study, a cohort study of European adults conducted over a similar period and with similar age distribution as CKB, showed that occupational physical activity was associated with lower risks of metabolic diseases, including colorectal cancer and liver cancer.^12 45^

Taken together, the consistency of the results over time and in diverse populations suggests that the inverse associations for occupational physical activity are likely to be real. A sensitivity analysis showed that an unmeasured confounder would have to be associated with physical activity and hepatobiliary diseases by a risk ratio of 1.6–3.0, above and beyond the measured confounders, to explain away the observed HR (online supplementary table 12), but weaker confounding could not do so.^46^ In addition, the associations remained for hepatobiliary diseases when excluding the first 5 years of follow-up or participants with chronic diseases or poor self-rated health at baseline, suggesting that the inverse associations were not affected by reverse causality.^47^

### The role of central adiposity

We found that additional adjustment for central adiposity partially attenuated the inverse associations of physical activity with chronic liver disease and gallstone disease. In line with our observation, a randomised controlled trial among patients with non-alcoholic fatty liver disease showed that vigorous and moderate exercise reduced intrahepatic triglyceride content and the effect was largely mediated by weight loss.^38^ For liver cancer, the EPIC study showed that waist circumference explained 40% of the inverse associations for total physical activity,^12^ whereas waist circumference explained little of the inverse association in CKB. This is possibly because a much smaller proportion of liver cancer is attributable to metabolic risk factors in China than in Western countries.^18^

### Mechanisms: how might physical activity affect the hepatobiliary system?

There are several potential mechanisms why physical activity is associated with lower risk of hepatobiliary cancers and diseases.^17 48^ First, higher physical activity is associated with lower risks of adiposity, hyperinsulinaemia and diabetes,^49 50^ which are associated with hepatobiliary cancers and diseases.^39 51–53^ Second, physical activity is associated with lower levels of systemic inflammation by altering proinflammatory and anti-inflammatory factors or adipokines (eg, C-reactive protein, adiponectin and interleukin-6),^54 55^ which are associated with higher risk of hepatobiliary cancers and diseases.^56 57^ Third, physical activity may have inverse associations by influencing the number and function of natural killer cells.^58 59^ Fourth, physical activity may influence risk of gallbladder disease by modulating gallbladder motility and increasing vagal tone and gastrointestinal transit time,^60^ which can promote bile stasis and crystal formation and decrease cholesterol resorption, leading to gallstones.^39^

### Strengths and limitations

The strengths of this CKB study include its prospective design, a large and diverse study population, and data on both occupational and non-occupational physical activity. We adjusted extensively for risk factors for hepatobiliary diseases.

We note at least four limitations. First, our study relied on hospital records to capture hepatobiliary diseases, which may have resulted in underestimation of the disease rates, particularly for non-alcoholic fatty liver disease, which is often ‘silent’. However, our risk estimates are unlikely to be greatly biased because (1) we ascertained all non-alcoholic fatty liver disease cases diagnosed between 2013 and 2015 and showed that 93% of all cases were diagnosed by ultrasound or CT; (2) our risk estimates agreed with previous studies of ultrasound-detected non-alcoholic fatty liver disease and symptomatic gallstone disease and our risk estimates were consistent across population subgroups (online supplementary figures 7, 8 and 12); (3) hospitalised non-alcoholic fatty liver disease and alcoholic liver disease have been shown to be valid in previous CKB reports on adiposity and diabetes;^53 61^ and (4) the misclassification is unlikely to differ by level of physical activity or other variables, which suggests that the association would be biased towards the null.

Second, the use of self-report may underestimate prevalent liver diseases at baseline, particularly for alcoholic liver disease and non-alcoholic fatty liver disease. For non-alcoholic fatty liver disease, we showed similar associations when excluding participants with elevated alanine transaminase (≥33 U/L for men and ≥25 U/L for women) in a nested case–control study of ~18 000 participants with blood biochemistry data (online supplementary table 13).

Third, although similar associations were observed when excluding the first 5 years of follow-up and when excluding participants with chronic diseases or poor self-rated health at baseline, reverse causality cannot be fully addressed because of the long latency period of cancer. Fourth, residual confounding may still exist due to unknown or unmeasured (eg, socioeconomic status, infections, inflammation and lipids) factors.

In summary, this large prospective study provides new evidence that higher levels of total physical activity, particularly occupational physical activity, were inversely associated with risk of major hepatobiliary diseases in Chinese adults, including non-alcoholic fatty liver disease, cirrhosis and cancer. The associations of total physical activity were consistent with previous prospective cohort studies conducted mostly in Western countries. For non-occupational physical activity (particularly leisure-time physical activity), more evidence is needed in populations with a diverse range of activities.

**Table 1:** Baseline characteristics of participants by level of total physical activity

Variable*	Total physical activity (MET-hours/day)				
	0–8.9	8.9–14.3	14.3–22.0	22.0–33.4	≥33.4
	(n=91 228)	(n=91 110)	(n=92 604)	(n=92 655)	(n=93 340)
Age (SD), years	58.4 (10.9)	54.7 (10.6)	50.5 (10.0)	48.5 (9.2)	47.1 (8.2)
Female, %	53.3	68.4	61.7	57.2	50.6
Socioeconomic and lifestyle factors					
Urban region, %	53.7	48.4	46.3	37.2	32.5
≥9 years of education, %	17.8	23.4	25.4	21.6	17.1
Household income ≥¥35 000/year, %	15.3	20.8	21.1	17.4	14.3
Ever regular smoking, %					
Male	69.3	67.9	65.9	67.3	67.7
Female	3.5	2.7	2.5	2.5	2.5
Weekly drinking, %					
Male	30.7	34.9	34.4	34.9	34.5
Female	1.9	2.0	2.1	2.3	2.4
Total physical activity (SD), MET-hours/day	5.9 (2.5)	11.8 (1.6)	17.8 (2.2)	27.1 (3.3)	42.1 (8.3)
Sedentary leisure time (SD), hours/day	3.6 (1.8)	3.3 (1.5)	3.0 (1.4)	2.7 (1.4)	2.6 (1.3)
Blood pressure and anthropometry					
SBP (SD), mm Hg	131.8 (23.2)	131.4 (22.0)	130.7 (20.7)	130.3 (20.2)	129.3 (19.4)
RPG (SD), mmol/L	6.2 (2.9)	6.1 (2.5)	6.0 (2.2)	6.0 (2.0)	5.9 (1.8)
BMI (SD), kg/m^2^	23.9 (3.6)	23.8 (3.5)	23.7 (3.3)	23.4 (3.2)	23.3 (3.1)
Waist circumference (SD), cm	81.2 (10.3)	80.9 (9.8)	80.3 (9.7)	79.5 (9.4)	78.9 (9.0)
Hip circumference (SD), cm	91.3 (7.5)	91.3 (7.1)	91.0 (6.8)	90.5 (6.4)	90.0 (6.1)
Waist to hip ratio (SD)	0.89 (0.07)	0.89 (0.07)	0.88 (0.07)	0.88 (0.07)	0.87 (0.07)
Per cent body fat (SD), %	28.6 (9.0)	28.4 (8.6)	27.8 (8.1)	27.3 (8.0)	26.8 (7.8)
Height (SD), cm	158.8 (8.3)	159.0 (8.0)	159.0 (8.4)	158.7 (8.4)	157.8 (7.9)
Disease history, %					
Diabetes	7.8	6.3	5.3	4.4	3.6
Coronary heart disease	3.5	3.1	2.6	2.1	1.5
Stroke or TIA	3.2	1.6	1.2	1.1	0.9
Hypertension	13.9	12.8	10.8	9.5	8.6
Family history of diabetes	4.8	5.3	5.3	5.0	4.8
Family history of cancer	13.7	13.8	14.0	14.3	13.6

*Results were standardised by age, sex and region (where appropriate). Values are means unless otherwise stated.

BMI, body mass index; MET, metabolic equivalent of task; RPG, random plasma glucose; SBP, systolic blood pressure; TIA, transient ischaemic attack.

**What are the findings?**

- In this Chinese population, total physical activity was inversely associated with a diverse range of hepatobiliary diseases, with similar patterns seen for occupational physical activity.
- The associations for non-occupational physical activity differed by disease subtype, with an inverse association for leisure-time physical activity with liver cancer and an inverse trend for gallstone disease.

**How might it impact on clinical practice in the future?**

- The benefits of higher levels of total physical activity are independent of lifestyle factors, including smoking, alcohol and sedentary leisure time, and are generally consistent across subtypes of hepatobiliary diseases.
- We identified that more evidence is needed in populations with a diverse range of activities to understand the effects of non-occupational physical activity on hepatobiliary health.

## Data Availability

Data are available upon reasonable request. The China Kadoorie Biobank (CKB) is a global resource for the investigation of lifestyle, environmental, blood biochemical and genetic factors as determinants of common diseases. The CKB study group is committed to making the cohort data available to the scientific community in China, the UK and worldwide to advance knowledge about the causes, prevention and treatment of disease. For detailed information on what is currently available to open access users, visit: http://www.ckbiobank.org/site/Data+Access. Researchers who are interested in obtaining the raw data from the CKB study that underline this paper should contact ckbaccess@ndph.ox.ac.uk. A research proposal will be requested to ensure that any analysis is performed by bona fide researchers and—where data are not currently available to open access researchers—is restricted to the topic covered in this paper.
