# Supplementary material for: Association of physical activity with risk of hepatobiliary diseases in China: a prospective cohort study of 0.5 million people
Source: Br J Sports Med. 2020 Aug 21;55(18):1024–33. doi: 10.1136/bjsports-2020-102174 (PMC8408581; doi:10.1136/bjsports-2020-102174)
Supplement: Supplementary data [file bjsports-2020-102174supp001.pdf]

Supplementary materials

Association of physical activity with risk of hepatobiliary diseases in Chinese: a prospective study of 0.5 million people

British Journal of Sports Medicine

Yuanjie Pang, DPhil[1], Jun Lv, PhD[1], Christiana Kartsonaki, DPhil[2,3], Canqing Yu, PhD[1], Yu Guo, MSc[4], Huaidong Du, PhD[2,3], Derrick Bennett, PhD[2,5], Zheng Bian, MSc[4], Yiping Chen, DPhil[2,3], Ling Yang, PhD[2,3], Iain Turnbull, MRCP[2], Hao Wang, MSc[6], Hui Li, MSc[7], Michael V. Holmes, PhD[2,3,5], Junshi Chen, MD[8], Zhengming Chen, DPhil[2], Liming Li, MD[1,4]

1. Department of Epidemiology & Biostatistics, School of Public Health, Peking University Health Science Centre, 38 Xueyuan Road, Beijing 100191, China
2. Clinical Trial Service Unit & Epidemiological Studies Unit (CTSU), Nuffield Department of Population Health, Big Data Institute Building, Roosevelt Drive, University of Oxford, UK
3. Medical Research Council Population Health Research Unit (MRC PHRU) at the University of Oxford, Nuffield Department of Population Health, University of Oxford, UK
4. Chinese Academy of Medical Sciences, 9 Dongdan San Tiao, Beijing 100730, China
5. National Institute for Health Research Oxford Biomedical Research Centre, Oxford University Hospital, Old Road, Oxford OX3 7LE, UK
6. Zhejiang Center for Disease Prevention and Control, 3399 Binsheng Road, Hangzhou 310051, China
7. Liuzhou Chinese Medicine Hospital, 32 Jiegangbei Road, Liuzhou 545001, China
8. National Center for Food Safety Risk Assessment, 37 Guangqu Road, Beijing 100021, China

Addresses for correspondence:

Prof. Zhengming Chen
CTSU
NDPH, Big Data Institute Building
University of Oxford
Old Road Campus
Oxford, OX3 7LF, UK
Tel: 44-1865-743839
Fax: 44-1865-743985
zhengming.chen@ndph.ox.ac.uk

or

Prof. Liming Li
Department of Epidemiology & Biostatistics
School of Public Health
Peking University Health Science Center
38 Xueyuan Road
Beijing, 100191, China
Tel: 86-010-82801528
Fax: 86-010-82801530
lmleeph@vip.163.com

Pang Y, *et al. Br J Sports Med* 2021; 55:1024–1033. doi: 10.1136/bjsports-2020-102174

BMJ Publishing Group Limited (BMJ) disclaims all liability and responsibility arising from any reliance placed on this supplemental material which has been supplied by the author(s)

Table of Contents

eMethods 3
Supplementary Table 1. Physical activity types, MET values, codes, and intensity categories ..8
Supplementary Table 2. Numbers of participants with hepatobiliary diseases in CKB 9
Supplementary Table 3. Estimated regression dilution ratios of physical activity (PA) 10
Supplementary Table 4. Mean and standard deviations (SDs) of PA by sex and regions 11
Supplementary Table 5. Associations of PA with risk of hepatobiliary diseases 12
Supplementary Table 6. Associations of PA with risk of hepatobiliary cancers by subtype 14
Supplementary Table 7. Associations of PA with risk of hepatobiliary diseases excluding the first five years of follow-up 15
Supplementary Table 8. Associations of baseline variables with risk of liver cancer and accidental death 16
Supplementary Table 9. Associations of PA with risk of liver cancer and accidental death 17
Supplementary Table 10. Percent change in the association of total PA with hepatobiliary diseases with additional adjustment for adiposity and diabetes 18
Supplementary Table 11. Associations of occupational PA with risk of hepatobiliary diseases by birth cohort 19
Supplementary Table 12. E-values for total PA and hepatobiliary diseases 20
Supplementary Table 13. Associations of total PA with hospitalised nonalcoholic fatty liver disease 21
Supplementary Figure 1. Literature search for the meta-analysis 22
Supplementary Figure 2. Associations of total PA with risk of hepatobiliary cancers and diseases 23
Supplementary Figure 3. Associations of occupational and nonoccupational PA with risk of liver diseases and cancer 24
Supplementary Figure 4. Associations of occupational and nonoccupational PA with risk of gallbladder diseases and GBTC 25
Supplementary Figure 5. Associations of domain-specific PA with risk of hepatobiliary disease by region 26
Supplementary Figure 6. Associations of domain-specific PA with risk of hepatobiliary disease by sex 27
Supplementary Figure 7. Associations of total PA with risk of chronic liver disease and liver cancer by participant characteristic 28
Supplementary Figure 8. Associations of total PA with risk of gallstone disease and GBTC by participant characteristic 29
Supplementary Figure 9. Associations of total PA with risk of hepatobiliary diseases with different exclusions 30
Supplementary Figure 10. Associations of domain-specific PA with risk of liver cancer and accidental death 31
Supplementary Figure 11. Meta-analysis of prospective studies on PA and hepatobiliary diseases 32
Supplementary Figure 12. Associations of total PA with risk of nonalcoholic fatty liver disease by participant characteristic 33

eMethods

Follow-up for morbidity and mortality

In CKB, C22 was used only for primary liver cancer and C22.9 was used for 'liver cancer, unspecified subsite'. Secondary cancer was coded as 'C78.7'. Information on cancer histological subtypes was also collected for a subset of the cases through cancer registries or reviews of hospital medical notes as part of the ongoing outcome adjudication for major diseases. Participants who had developed hepatobiliary cancers and diseases were censored at the first time of event. Participants who died or were lost to follow-up were censored at the last day known to be alive.

Statistical analysis

A Cox proportional hazards models with age as the underlying time scale and delayed entry at age at baseline were used to estimate adjusted hazard ratios (HRs) of specific disease incidence associated with physical activity levels, stratified by sex and study area (10 areas), and adjusted for age at baseline, education (6 groups: no formal school, primary school, middle school, high school, technical school/college, or university), household income (6 groups: <2500, 2500-4999, 5000-9999, 10,000-19,999, 20,000-34,999, or ≥35,000 RMB), smoking (3 groups: never, occasional, or ever regular), alcohol (6 groups: abstainers, ex-weekly drinkers, reduced-intake drinkers, occasional drinkers, and among weekly drinkers, <20 or ≥20 g/day [women], <30 or ≥30 g/day [men]), self-rated health (3 groups: poor, fair, and good), diabetes, cardiovascular disease, respiratory disease, rheumatoid arthritis, and sedentary leisure time. These confounders were selected based on a literature review and previous reports in CKB and included potential risk factors for

hepatobiliary diseases (sex, age, smoking, alcohol, self-rated health, diabetes, and sedentary leisure time)[1-4,5] and factors associated with physical activity (sex, age, smoking, alcohol, self-rated health, prior history of diabetes, cardiovascular disease, respiratory disease, and rheumatoid arthritis).[6-10] In CKB, risks of hepatobiliary diseases and patterns of physical activity varied across 10 regions, so all analyses were stratified by study region.[12,13] Education and household income were included to reflect socioeconomic status associated with both physical activity and hepatobiliary diseases.[13] Physical activity was categorised by splitting at quintiles in order to assess the shape of the association. If the association was linear then physical activity was also modelled as a continuous variable to estimate risk associated with a 4 MET-h/day higher level of physical activity.

To assess potentially nonlinear associations between physical activity and disease risk, restricted cubic splines were calculated using three fixed knots at the 10%, 50%, and 90% quintiles. Nonlinearity was evaluated using the likelihood ratio rest to compare the fit of linear and nonlinear models.

For exposure variables with more than two categories, all HRs are presented with ‘floating’ standard errors to facilitate comparisons between groups.[14] The CKB estimates for physical activity and hepatobiliary cancers were meta-analysed with estimates from published prospective cohort studies using a random effects meta-analysis. Details of the study selection are reported in **Supplementary Figure 1**.

Single measurements of physical activity may not accurately reflect an individual’s usual level because of within-person variation or change over time. Repeat measurements of physical activity were available for ~20,000 participants who attended a resurvey ~3 years

after baseline and were used to estimate regression dilution ratios (RDRs) using the McMahon-Peto method (**Supplementary Table 3**).[15] Log HR estimates for 4 MET-h/day higher physical activity were divided by the RDR to estimate associations of usual physical activity with incident disease risk.

We selected *a priori* central adiposity (waist circumference) and diabetes and investigated the extent to which additional adjustment for these factors could alter the association of physical activity with hepatobiliary diseases. We included each of the above-identified factors in the basic model and examined the percent change in the logHRs comparing the top and bottom quintile of total physical activity. The proportion of disease risk reduction due to additional adjustment for the factor was calculated as follows: $((logHR_{adjusted\ model} - logHR_{basic\ model}) / (logHR_{basic\ model})) \times 100\%$. The 95% CIs for the proportion were obtained through bootstrap with 1000 replications.

Sensitivity analyses

We conducted several sensitivity analyses. First, previous reports showed that the patterns of physical activity differed by sex and region in CKB,[11] and therefore we examined whether the associations of domain-specific physical activity with hepatobiliary diseases differ by sex and urbanicity. Importantly, urbanicity is a major source of confounding by socioeconomic status and other unmeasured confounders (e.g. infections), which are associated with chronic liver disease and liver cancer.[13,15] Second, we explored whether the association of total physical activity with hepatobiliary diseases differed in subgroups defined by age, sex, region, education, smoking, alcohol, obesity, diabetes, hypertension, sedentary leisure time, and work activity intensity (farmers, manual workers, and

nonmanual workers). Work activity intensity was used to assess whether the associations differed by the intensity of physical activity (the mean MET values of total physical activity in CKB: farmers 10.4, manual workers 11.7, and nonmanual workers 6.4). Third, the main analyses were repeated excluding the first five years of follow-up. This is because participants with subclinical or undetected diseases at baseline might be diagnosed with disease over early years of follow-up and subclinical diseases may affect physical activity at baseline, resulting in reverse causation bias.[6-10] Fourth, the main analyses were repeated excluding participants with poor self-rated health status or major chronic diseases (including cardiovascular disease [CVD], tuberculosis, respiratory disease, peptic ulcer, and kidney disease). Poor self-rated health or major chronic diseases may affect physical activity, which may result in reverse causation bias. Fifth, we used accidents as a negative control outcome to explore potential bias and confounding in the associations of physical activity with hepatobiliary diseases.[17]

References

1. Stinton LM, Shaffer EA. Epidemiology of gallbladder disease: cholelithiasis and cancer. *Gut Liver* 2012;6:172-87.

2. Word Cancer Research Fund/American Insisute for Cancer Research. Continuous Update Project Expert Report 2018. Diet, nutrition, physical activity and liver cancer. Available at dietandcancerreport.org.

3. Word Cancer Research Fund/American Insisute for Cancer Research. Continuous Update Project Expert Report 2018. Diet, nutrition, physical activity and gallbladder cancer. Available at dietandcancerreport.org.

4. Tsochatzis EA, Bosch J, Burroughs AK. Liver cirrhosis. *Lancet* 2014;383:1749-61.

5. Shaffer EA. Gallstone disease: Epidemiology of gallbladder stone disease. *Best Pract Res Clin Gastroenterol* 2006;20:981-96.

6. Behrens G, Leitzmann MF. The association between physical activity and renal cancer: systematic review and meta-analysis. *Br J Cancer* 2013;108:798-811.

7. Baumeister SE, Schlesinger S, Aleksandrova K, Jochem C, Jenab M, Gunter MJ, et al. Association between physical activity and risk of hepatobiliary cancers: A multinational cohort study. *J Hepatol* 2019;70:885-92.

8. Moore SC, Lee IM, Weiderpass E, Campbell PT, Sampson JN, Kitahara CM, et al. Association of leisure-time physical activity with risk of 26 types of cancer in 1.44 million adults. *JAMA Intern Med* 2016;176:816-25.

9. Qiu S, Cai X, Sun Z, Li L, Zügel M, Steinacker JM, et al. Association between physical activity and risk of nonalcoholic fatty liver disease: a meta-analysis. *Therap Adv Gastroenterol* 2017;10:701-13.

10. Aune D, Leitzmann M, Vatten LJ. Physical activity and the risk of gallbladder disease: a systematic review and meta-analysis of cohort studies. *J Phys Act Health* 2016;13:788-95.

11. Du H, Bennett D, Li L, Whitlock G, Guo Y, Collins R, et al. Physical activity and sedentary leisure time and their associations with BMI, waist circumference, and percentage body fat in 0.5 million adults: the China Kadoorie Biobank study. *Am J Clin Nutr* 2013;97:487-96.

12. Pang Y, Kartsonaki C, Turnbull I, Guo Y, Clarke R, Chen Y, et al. Diabetes, plasma glucose, and incidence of fatty liver, cirrhosis, and liver cancer: a prospective study of 0.5 million people. *Hepatology* 2018;68:1308-18

13. Pang Y, Kartsonaki C, Guo Y, Chen Y, Yang L, Bian Z, et al. Socioeconomic status in relation to risks of major gastrointestinal cancers in Chinese adults: a prospective study of 0.5 million people. *Cancer Epidemiol Biomarkers Prev* 2020; 29(4): 823-31.

14. Easton DF, Peto J, Babiker AG. Floating absolute risk: an alternative to relative risk in survival and case-control analysis avoiding an arbitrary reference group. *Stat Med* 1991;10:1025-35.

15. MacMahon S, Peto R, Cutler J, Collins R, Sorlie P, Neaton J, et al. Blood pressure, stroke, and coronary heart disease. Part 1, Prolonged differences in blood pressure: prospective observational studies corrected for the regression dilution bias. *Lancet* 1990;335:765-74.

16. Wang FS, Fan JG, Zhang Z, Gao B, Wang HY. The global burden of liver disease: the major impact of China. *Hepatology* 2014;60:2099-108.

17. Hamer M, Bauman A, Bell JA, Stamatakis E. Examining associations between physical activity and cardiovascular mortality using negative control outcomes. Int J Epidemiol 2019; 48(4): 1161-1166.

Supplementary Table 1. Physical activity types, MET values, codes, and intensity categories

Activity type	MET value	Intensity	Code
Sedentary work	1.8	Low	Mean of 11580, 11585, and 11590
Standing work	3.8	Moderate	Mean of 11610 and 11630
Manual work	4.5	Moderate	11476
Heavy manual work	6.5	Vigorous	11477
Work outside the farming season	2.0	Low	11147
Fully mechanised work in the farming season	2.4	Low	Mean of 11147 and 11170
Semi-mechanised work in the farming season	3.4	Moderate	Mean of 11146 and 11147
Manual work in the farming season	6.3	Vigorous	Mean of 11145 and 11146
Walking	4.0	Moderate	17270
Bicycle	6.8	Vigorous	1011
Motorbike	3.5	Moderate	16030
Private or public transportation	1.7	Low	Mean of 16010, 16015, and 16016
Household activity	2.8	Low	Mean of 05030[*], 05040[*], 05035, 05055, 05070, 05090[*], 05092[*], 05184, 05197, and 05200
Tai-Chi/qigong/leisure walking	3.3	Moderate	Mean of 15670 and 17160
Jogging/aerobic exercise	7.4	Vigorous	Mean of 03015, 12020, and 12150
Ball games	5.5	Moderate	Mean of 15020[*], 15030[*], 15055, 15080, 15090, 15255, 15605[*], 15610[*], 15652, 15660, 15675, 15710[*], and 15711[*]
Brisk walking/gymnastics/folk dancing	4.2	Moderate	Mean of 03025, 15300, and 17200
Swimming	7.2	Vigorous	Mean of 18230, 18240, and 18310
Other exercise, e.g. mountain walking, home exercise and rope jumping	5.9	Moderate	Mean of 02010, 02064, 04001, 04100, 15110[*], 15120[*], 15200, 15240, 15310, 15425[*], 15430[*], 15537, 15550[†], 15551[†], 15552[†], 15580, 15590, 15730, 15732[†], 15733[†], 15734[†], and 19030

This table is adapted from the supplementary materials by Du et al. (2015) with the citation "Du H, Bennett D, Li L, Whitlock G, Guo Y, Collins R, et al. Physical activity and sedentary leisure time and their associations with BMI, waist circumference, and percentage body fat in 0.5 million adults: the China Kadoorie Biobank study. *Am J Clin Nutr.* 2013; 97: 487-96.".
Abbreviations: MET, Metabolic equivalent of tasks.
[*] Assigned 1/2 weight in calculating the mean MET value because the connecting two items represent one type of activity.
[†] Assigned 1/3 weight in calculating the mean MET value because the connecting three items represent one type of activity.
A quality control survey in CKB involving 923 participants with repeated measurements within one year after the baseline survey showed reasonable reliability of exercise participation (kappa=0.60) and total physical activity (intraclass correlation coefficient [ICC]=0.72), which is comparable to EPIC and SWHS (ICC for total physical activity: EPIC men 0.75, EPIC women 0.68, and SWHS 0.70; kappa for exercise participation: SWHS 0.64) (references: Matthews CE, Shu XO, Yang G, Jin F, Ainsworth BE, Liu D, et al. Reproducibility and validity of the Shanghai Women's Health Study physical activity questionnaire. *Am J Epidemiol* 2003;158:1114-22. Pols MA, Peeters PH, Ocke MC, Slimani N, Bueno-de-Mesquita HB, Collette HJ. Estimation of reproducibility and relative validity of the questions included in the EPIC Physical Activity Questionnaire. *Int J Epidemiol* 1997;26(Suppl 1):S181-9).

Supplementary Table 2. Numbers of participants with hepatobiliary diseases in CKB

Description	ICD-10	No. of cases			
		Total	Health insurance	Death registry	Disease registry
Chronic liver disease					
Alcoholic liver disease	K70	**311**	311	0	0
Cirrhosis	K74	**2511**	1629	731	10
Nonalcoholic fatty liver disease	K76.0	**1498**	1498	0	0
Chronic viral hepatitis	B18	**993**	443	368	2
Unspecified viral hepatitis	B19	**988**	785	28	0
**Total**		**5285**			
Liver cancer					
Primary liver cancer	C22	**2909**	1202	1874	955
Hepatocellular carcinoma	C22.0	**295**	70	100	99
Intrahepatic bile duct cancer	C22.1	**165**	44	82	54
Gallbladder disease					
Cholelithiasis	K80	**11,170**	8473	46	14
Cholecystitis	K81	**10,390**	8431	22	6
**Total**		**19,638**			
Gallbladder and biliary tract cancer					
Gallbladder cancer	C23	**312**	133	104	100
Total bile duct cancer	C24	**402**	264	100	68
Extrahepatic bile duct cancer	C24.0	**322**	231	50	36

Pang Y, *et al. Br J Sports Med* 2021; 55:1024–1033. doi: 10.1136/bjsports-2020-102174

Supplementary Table 3. Estimated regression dilution ratios of physical activity (PA)

Baseline groups	Mean baseline value	Mean resurvey value	Ratio of the ranges estimate	Self-correlation estimate
**Total PA (MET-h/day)**				
<8.7 (1)	5.71	11.50	0.54	0.51
8.8 to <14.2 (2)	11.59	14.66		
14.3 to <21.8 (3)	17.71	18.82		
21.9 to <33.1 (4)	27.32	25.06		
≥33.2 (5)	43.23	31.67		
Difference (5 - 1)	***37.52***	***20.17***		
**Occupational PA (MET-h/day)**				
0 (1)	0	2.25	0.62	0.57
0.1 to <9.0 (2)	4.57	9.37		
9.0 to <15.5 (3)	11.85	13.53		
15.6 to <26.1 (4)	20.98	18.46		
≥26.2 (5)	37.42	25.44		
Difference (5 - 1)	***37.42***	***23.19***		
**Nonoccupational PA (MET-h/day)**				
<3.9 (1)	2.11	4.54	0.49	0.48
3.9 to <6.4 (2)	5.30	6.74		
6.5 to <8.4 (3)	7.71	8.23		
8.5 to <11.6 (4)	10.10	9.21		
≥11.7 (5)	15.53	11.14		
Difference (5 - 1)	***13.42***	***6.60***		

Pang Y, *et al. Br J Sports Med* 2021; 55:1024–1033. doi: 10.1136/bjsports-2020-102174

Supplementary Table 4. Mean and standard deviations (SDs) of PA by sex and regions

Domain	Urban			Rural		
	Overall	Men	Women	Overall	Men	Women
**Physical activity (MET-h/day)**						
Total	18.7 (12.7)	19.4 (13.9)	17.9 (11.7)	23.2 (14.4)	24.8 (16.0)	22.0 (13.2)
Occupational	10.4 (12.9)	13.4 (13.7)	8.3 (11.9)	15.0 (14.3)	1.95 (15.6)	11.9 (12.6)
Nonoccupational	8.1 (4.9)	5.9 (4.2)	9.6 (4.7)	8.2 (5.0)	5.3 (4.0)	10.2 (4.6)
Commuting	1.1 (1.9)	1.4 (2.0)	0.9 (1.7)	1.9 (2.5)	2.2 (2.6)	1.7 (2.4)
Household	5.3 (4.1)	2.8 (2.6)	7.0 (4.0)	6.1 (4.3)	2.9 (2.9)	8.3 (3.7)
Leisure-time	1.7 (3.0)	1.8 (3.1)	1.7 (2.9)	0.2 (1.1)	0.3 (1.2)	0.2 (0.9)

Supplementary Table 5. Associations of PA with risk of hepatobiliary diseases

	Alcoholic liver disease		Nonalcoholic fatty liver disease		Viral hepatitis		Cirrhosis		Liver cancer	
	No. cases	HR (95% CI)	No. cases	HR (95% CI)	No. cases	HR (95% CI)	No. cases	HR (95% CI)	No. cases	HR (95% CI)
**PA (MET-h/day)**										
<8.7	59	1.00 (0.74, 1.35)	241	1.00 (0.87, 1.15)	222	1.00 (0.86, 1.17)	414	1.00 (0.89, 1.12)	635	1.00 (0.91, 1.10)
8.8 to 14.2	41	0.71 (0.52, 0.96)	255	0.93 (0.82, 1.06)	186	0.85 (0.73, 0.99)	322	0.93 (0.83, 1.04)	411	0.92 (0.83, 1.01)
14.3 to 21.8	52	0.80 (0.61, 1.05)	343	0.90 (0.81, 1.00)	187	0.83 (0.72, 0.96)	290	0.87 (0.77, 0.97)	355	0.92 (0.83, 1.02)
21.9 to 33.1	53	0.84 (0.63, 1.10)	305	0.82 (0.73, 0.92)	195	0.82 (0.71, 0.96)	338	0.94 (0.83, 1.05)	357	1.00 (0.89, 1.12)
≥33.2	59	0.92 (0.70, 1.22)	232	0.62 (0.53, 0.72)	164	0.73 (0.62, 0.87)	238	0.76 (0.66, 0.88)	261	0.81 (0.71, 0.93)
per 4 units		*1.01 (0.93, 1.09)*		*0.90 (0.87, 0.94)*		*0.95 (0.91, 0.99)*		*0.94 (0.91, 0.98)*		*0.96 (0.93, 0.99)*
**Occupational PA**										
0	54	1.00 (0.69, 1.45)	348	1.00 (0.86, 1.16)	308	1.00 (0.84, 1.19)	584	1.00 (0.88, 1.13)	841	1.00 (0.90, 1.12)
0.1 to <9.0	57	0.81 (0.60, 1.08)	268	1.05 (0.93, 1.20)	180	0.83 (0.70, 0.99)	295	0.95 (0.83, 1.08)	359	0.92 (0.82, 1.04)
9.0 to <15.5	39	0.62 (0.45, 0.85)	318	0.97 (0.87, 1.09)	152	0.70 (0.59, 0.82)	239	0.75 (0.66, 0.85)	285	0.90 (0.80, 1.01)
15.6 to <26.1	58	0.90 (0.69, 1.17)	220	0.78 (0.68, 0.89)	185	0.84 (0.72, 0.97)	302	0.92 (0.82, 1.04)	304	0.99 (0.88, 1.12)
≥26.2	56	0.77 (0.58, 1.03)	222	0.69 (0.59, 0.81)	129	0.65 (0.54, 0.79)	182	0.66 (0.56, 0.77)	230	0.78 (0.68, 0.90)
per 4 units		*1.00 (0.93, 1.07)*		*0.91 (0.88, 0.94)*		*0.95 (0.91, 0.99)*		*0.93 (0.90, 0.97)*		*0.96 (0.94, 0.99)*
**Nonoccupational PA**										
<3.9	85	1.00 (0.80, 1.25)	270	1.00 (0.87, 1.15)	209	1.00 (0.85, 1.18)	316	1.00 (0.88, 1.13)	482	1.00 (0.90, 1.11)
3.9 to <6.4	79	1.27 (1.02, 1.58)	285	1.12 (1.00, 1.26)	190	1.00 (0.86, 1.15)	315	1.10 (0.98, 1.23)	434	1.07 (0.97, 1.18)
6.5 to <8.4	40	1.03 (0.75, 1.41)	288	1.17 (1.05, 1.32)	188	0.98 (0.85, 1.13)	326	1.18 (1.06, 1.31)	385	1.02 (0.92, 1.12)
8.5 to <11.6	33	1.14 (0.80, 1.63)	284	0.99 (0.88, 1.12)	167	0.88 (0.75, 1.03)	306	1.15 (1.02, 1.29)	360	0.99 (0.89, 1.11)
≥11.7	27	1.87 (1.25, 2.80)	249	1.06 (0.93, 1.22)	200	1.00 (0.85, 1.17)	339	1.20 (1.06, 1.36)	358	1.07 (0.96, 1.20)
per 4 units		*1.14 (0.86, 1.50)*		*0.97 (0.86, 1.08)*		*1.00 (0.88, 1.14)*		*1.04 (0.95, 1.15)*		*0.98 (0.90, 1.07)*

Models were stratified by sex and region, and adjusted for age at baseline, education, household income, smoking, alcohol, self-rated health, diabetes, cardiovascular disease, respiratory disease, rheumatoid arthritis, and sedentary leisure time.

Supplementary Table 5. continued

	Gallstone disease		Cholecystitis		Gallbladder cancer		Biliary tract cancer	
	No. cases	HR (95% CI)	No. cases	HR (95% CI)	No. cases	HR (95% CI)	No. cases	HR (95% CI)
**PA (MET-h/day)**								
<8.7	1535	1.00 (0.94, 1.06)	1343	1.00 (0.94, 1.07)	63	1.00 (0.75, 1.33)	107	1.00 (0.80, 1.25)
8.8 to 14.2	1530	0.94 (0.89, 0.99)	1244	0.96 (0.90, 1.01)	50	1.05 (0.79, 1.40)	76	1.01 (0.80, 1.28)
14.3 to 21.8	1756	0.90 (0.86, 0.95)	1472	1.00 (0.95, 1.05)	46	1.08 (0.81, 1.44)	61	0.90 (0.70, 1.16)
21.9 to 33.1	1900	0.87 (0.83, 0.91)	2206	1.05 (1.01, 1.10)	52	1.26 (0.94, 1.70)	55	0.84 (0.63, 1.12)
≥33.2	1852	0.86 (0.81, 0.90)	2265	1.06 (1.01, 1.11)	22	0.51 (0.32, 0.80)	37	0.55 (0.38, 0.78)
per 4 units		*0.98 (0.96, 0.99)*		*1.01 (1.00, 1.03)*		*0.92 (0.84, 1.01)*		*0.91 (0.84, 0.98)*
**Occupational PA**								
0	2426	1.00 (0.95, 1.06)	2022	1.00 (0.94, 1.06)	95	1.00 (0.74, 1.35)	148	1.00 (0.76, 1.31)
0.1 to <9.0	1512	0.90 (0.85, 0.95)	1298	1.17 (1.10, 1.24)	42	0.99 (0.70, 1.40)	50	0.88 (0.65, 1.20)
9.0 to <15.5	1532	0.89 (0.85, 0.94)	1596	1.11 (1.06, 1.17)	33	0.95 (0.68, 1.34)	52	1.03 (0.78, 1.36)
15.6 to <26.1	1561	0.87 (0.83, 0.92)	1958	1.17 (1.12, 1.23)	38	1.05 (0.76, 1.46)	51	0.98 (0.74, 1.30)
≥26.2	1542	0.87 (0.82, 0.92)	1656	1.14 (1.08, 1.21)	25	0.62 (0.41, 0.95)	35	0.62 (0.43, 0.89)
per 4 units		*0.98 (0.97, 0.99)*		*1.01 (1.00, 1.03)*		*0.94 (0.86, 1.02)*		*0.92 (0.85, 1.00)*
**Nonoccupational PA**								
<3.9	1339	1.00 (0.94, 1.06)	1313	1.00 (0.94, 1.07)	49	1.00 (0.72, 1.39)	75	1.00 (0.77, 1.30)
3.9 to <6.4	1524	0.95 (0.91, 1.01)	1440	0.97 (0.92, 1.03)	43	0.81 (0.60, 1.09)	69	1.04 (0.82, 1.32)
6.5 to <8.4	1761	0.95 (0.91, 0.99)	1660	1.03 (0.98, 1.08)	59	1.06 (0.82, 1.37)	78	1.33 (1.06, 1.66)
8.5 to <11.6	2071	0.98 (0.93, 1.02)	1716	0.98 (0.93, 1.02)	46	0.88 (0.65, 1.18)	63	1.10 (0.85, 1.41)
≥11.7	1878	0.91 (0.86, 0.95)	2401	0.99 (0.94, 1.04)	36	0.70 (0.49, 0.99)	51	0.91 (0.67, 1.23)
per 4 units		*0.97 (0.93, 1.02)*		*1.01 (0.97, 1.05)*		*0.86 (0.66, 1.12)*		*0.89 (0.71, 1.11)*

Supplementary Table 6. Associations of PA with risk of hepatobiliary cancers by subtype

	Liver cancer		IHBC		Biliary tract cancer		EHBC	
	No. cases	HR (95% CI)	No. cases	HR (95% CI)	No. cases	HR (95% CI)	No. cases	HR (95% CI)
**PA (MET-h/day)**								
<8.7	635	1.00 (0.91, 1.10)	46	1.00 (0.71, 1.40)	107	1.00 (0.80, 1.25)	98	1.00 (0.79, 1.27)
8.8 to 14.2	411	0.92 (0.83, 1.01)	37	1.07 (0.76, 1.50)	76	1.01 (0.80, 1.28)	62	0.91 (0.70, 1.18)
14.3 to 21.8	355	0.92 (0.83, 1.02)	20	0.68 (0.44, 1.06)	61	0.90 (0.70, 1.16)	51	0.89 (0.68, 1.17)
21.9 to 33.1	357	1.00 (0.89, 1.12)	27	1.07 (0.70, 1.62)	55	0.84 (0.63, 1.12)	53	0.96 (0.71, 1.29)
≥33.2	261	0.81 (0.71, 0.93)	17	0.82 (0.49, 1.40)	37	0.55 (0.38, 0.78)	34	0.61 (0.42, 0.88)
per 4 units		*0.96 (0.93, 0.99)*		*0.99 (0.87, 1.12)*		*0.91 (0.84, 0.98)*		*0.93 (0.85, 1.01)*
**Occupational PA**								
0	841	1.00 (0.90, 1.12)	71	1.00 (0.66, 1.52)	148	1.00 (0.76, 1.31)	138	1.00 (0.75, 1.33)
0.1 to <9.0	359	0.92 (0.82, 1.04)	19	1.00 (0.61, 1.64)	50	0.88 (0.65, 1.20)	39	0.81 (0.57, 1.15)
9.0 to <15.5	285	0.90 (0.80, 1.01)	18	0.91 (0.57, 1.44)	52	1.03 (0.78, 1.36)	41	0.96 (0.70, 1.30)
15.6 to <26.1	304	0.99 (0.88, 1.12)	26	1.39 (0.93, 2.07)	51	0.98 (0.74, 1.30)	46	1.03 (0.77, 1.39)
≥26.2	230	0.78 (0.68, 0.90)	13	0.85 (0.48, 1.51)	35	0.62 (0.43, 0.89)	34	0.73 (0.51, 1.06)
per 4 units		*0.96 (0.94, 0.99)*		*0.90 (0.64, 1.25)*		*0.92 (0.85, 1.00)*		*0.92 (0.73, 1.16)*
**Nonoccupational PA**								
<3.9	482	1.00 (0.90, 1.11)	27	1.00 (0.65, 1.53)	75	1.00 (0.77, 1.30)	64	1.00 (0.75, 1.33)
3.9 to <6.4	434	1.07 (0.97, 1.18)	34	1.22 (0.86, 1.71)	69	1.04 (0.82, 1.32)	58	1.02 (0.79, 1.32)
6.5 to <8.4	385	1.02 (0.92, 1.12)	24	0.90 (0.60, 1.34)	78	1.33 (1.06, 1.66)	72	1.43 (1.14, 1.81)
8.5 to <11.6	360	0.99 (0.89, 1.11)	31	1.09 (0.76, 1.56)	63	1.10 (0.85, 1.41)	52	1.04 (0.79, 1.38)
≥11.7	358	1.07 (0.96, 1.20)	31	1.06 (0.71, 1.57)	51	0.91 (0.67, 1.23)	52	0.99 (0.73, 1.34)
per 4 units		*0.98 (0.90, 1.07)*		*1.00 (0.89, 1.13)*		*0.89 (0.71, 1.11)*		*0.94 (0.87, 1.02)*

Abbreviations: IHBC, intrahepatic bile duct cancer; EHBC, extrahepatic bile duct cancer.
Models were stratified by stratified by sex and region, and adjusted for age at baseline, education, household income, smoking, alcohol, self-rated health, diabetes, cardiovascular disease, respiratory disease, rheumatoid arthritis, and sedentary leisure time.

Supplementary Table 7. Associations of PA with risk of hepatobiliary diseases excluding the first five years of follow-up

	Chronic liver disease		Liver cancer		Gallstone disease		GBTC	
	No. cases	HR (95% CI)	No. cases	HR (95% CI)	No. cases	HR (95% CI)	No. cases	HR (95% CI)
**PA (MET-h/day)**								
<8.7	477	1.00 (0.90, 1.11)	359	1.00 (0.88, 1.13)	1135	1.00 (0.93, 1.08)	100	1.00 (0.78, 1.28)
8.8 to 14.2	452	0.93 (0.85, 1.02)	228	0.86 (0.75, 0.99)	1171	0.96 (0.90, 1.03)	79	1.13 (0.89, 1.43)
14.3 to 21.8	490	0.88 (0.81, 0.97)	197	0.86 (0.75, 0.99)	1265	0.88 (0.83, 0.94)	72	1.13 (0.89, 1.44)
21.9 to 33.1	493	0.90 (0.82, 0.99)	205	0.99 (0.86, 1.15)	1389	0.87 (0.81, 0.92)	66	0.99 (0.74, 1.32)
≥33.2	313	0.78 (0.70, 0.88)	143	0.77 (0.64, 0.92)	1279	0.87 (0.82, 0.94)	41	0.53 (0.36, 0.78)
per 4 units		*0.95 (0.92, 0.98)*		*0.97 (0.93, 1.01)*		*0.98 (0.96, 1.00)*		*0.91 (0.83, 0.99)*
**Occupational PA**								
Quintile 1	679	1.00 (0.89, 1.12)	476	1.00 (0.86, 1.16)	1845	1.00 (0.93, 1.07)	155	1.00 (0.76, 1.31)
Quintile 2	472	0.91 (0.82, 1.00)	193	0.90 (0.77, 1.06)	1104	0.87 (0.81, 0.93)	53	0.90 (0.65, 1.23)
Quintile 3	427	0.78 (0.71, 0.86)	163	0.90 (0.77, 1.05)	1162	0.85 (0.80, 0.91)	61	1.12 (0.86, 1.46)
Quintile 4	395	0.81 (0.73, 0.90)	175	1.03 (0.88, 1.20)	1102	0.85 (0.79, 0.90)	51	0.92 (0.68, 1.24)
Quintile 5	252	0.73 (0.64, 0.83)	125	0.78 (0.64, 0.94)	1026	0.87 (0.80, 0.94)	38	0.61 (0.42, 0.90)
per 4 units		*0.94 (0.92, 0.97)*		*0.97 (0.93, 1.01)*		*0.98 (0.97, 1.00)*		*0.92 (0.85, 1.00)*
**Nonoccupational PA**								
Quintile 1	434	1.00 (0.90, 1.12)	269	1.00 (0.87, 1.15)	932	1.00 (0.92, 1.08)	68	1.00 (0.75, 1.33)
Quintile 2	410	1.03 (0.93, 1.14)	247	1.07 (0.94, 1.22)	1087	0.91 (0.85, 0.98)	67	0.99 (0.77, 1.27)
Quintile 3	451	1.09 (0.99, 1.20)	217	1.00 (0.87, 1.14)	1222	0.87 (0.82, 0.93)	87	1.22 (0.97, 1.53)
Quintile 4	470	1.01 (0.92, 1.11)	192	0.90 (0.78, 1.05)	1541	0.93 (0.88, 0.99)	67	1.02 (0.79, 1.33)
Quintile 5	460	1.05 (0.94, 1.16)	207	1.00 (0.86, 1.17)	1457	0.87 (0.82, 0.93)	69	0.84 (0.62, 1.14)
per 4 units		*1.02 (0.94, 1.12)*		*0.95 (0.84, 1.07)*		*0.98 (0.93, 1.04)*		*0.89 (0.71, 1.12)*

Models were stratified by stratified by sex and region, and adjusted for age at baseline, education, household income, smoking, alcohol, self-rated health, diabetes, cardiovascular disease, respiratory disease, rheumatoid arthritis, and sedentary leisure time.

Pang Y, *et al. Br J Sports Med* 2021; 55:1024–1033. doi: 10.1136/bjsports-2020-102174

Supplementary Table 8. Associations of baseline variables with risk of liver cancer and accidental death

Variables	Liver cancer HR (95% CI)	Accidental death HR (95% CI)
**Age, per year**	1.01 (1.00, 1.03)	0.98 (0.97, 0.99)
**Female sex**	0.45 (0.40, 0.52)	0.48 (0.44, 0.55)
**Education, years**		
7-12 vs ≤6	0.90 (0.81, 0.99)	0.81 (0.75, 0.88)
≥13 vs ≤6	0.78 (0.63, 0.95)	0.70 (0.57, 0.86)
**Income, RMB**		
5000-20,000 vs <5000	0.93 (0.83, 1.05)	0.85 (0.78, 0.92)
≥20,000 vs <5000	0.84 (0.73, 0.96)	0.72 (0.65, 0.80)
**Self-rated health**		
Good vs excellent	0.95 (0.84, 1.08)	0.97 (0.88, 1.07)
Fair vs excellent	1.21 (1.08, 1.36)	1.18 (1.08, 1.30)
Poor vs excellent	1.61 (1.40, 1.84)	1.38 (1.23, 1.55)
**Smoking among men**		
Ex-regular vs never smoker	1.22 (0.94, 1.59)	1.09 (0.87, 1.37)
Current regular vs never smoker	1.26 (1.07, 1.48)	1.11 (0.98, 1.27)
**Alcohol among men**		
0-139 g/week vs non-drinker	0.83 (0.70, 1.00)	0.82 (0.70, 0.96)
140-419 g/week vs non-drinker	1.01 (0.85, 1.19)	1.18 (0.70, 1.34)
≥420 g/week vs non-drinker	1.36 (1.11, 1.66)	1.30 (1.03, 1.34)
**HBsAg, positive vs negative**	12.83 (11.76, 14.00)	1.63 (1.22, 2.19)

Models were adjusted for age at baseline, sex, region, education, household income, smoking, alcohol, self-rated health, and HBsAg, where appropriate. Participants with cancer at baseline were excluded. The analysis of smoking and alcohol was conducted in men because of the small amount of women who smoked or drank (2.4% current regular smokers and 2.1% weekly drinkers).

Supplementary Table 9. Associations of PA with risk of liver cancer and accidental death

	Liver cancer		Accidental death	
	**No. cases**	**HR (95% CI)**	**No. cases**	**HR (95% CI)**
**PA (MET-h/day)**				
<8.7	635	1.00 (0.91, 1.10)	1099	1.00 (0.93, 1.07)
8.8 to 14.2	411	0.92 (0.83, 1.01)	739	0.81 (0.75, 0.87)
14.3 to 21.8	355	0.92 (0.83, 1.02)	798	0.91 (0.85, 0.97)
21.9 to 33.1	357	1.00 (0.89, 1.12)	844	0.95 (0.88, 1.02)
≥33.2	261	0.81 (0.71, 0.93)	894	1.00 (0.93, 1.08)
per 4 units		*0.96 (0.93, 0.99)*		*1.01 (0.99, 1.03)*
**Occupational PA**				
Quintile 1	841	1.00 (0.90, 1.12)	1176	1.00 (0.92, 1.09)
Quintile 2	359	0.92 (0.82, 1.04)	976	0.90 (0.84, 0.97)
Quintile 3	285	0.90 (0.80, 1.01)	642	0.83 (0.77, 0.90)
Quintile 4	304	0.99 (0.88, 1.12)	749	0.96 (0.89, 1.03)
Quintile 5	230	0.78 (0.68, 0.90)	831	0.98 (0.91, 1.06)
per 4 units		*0.96 (0.94, 0.99)*		*1.01 (0.99, 1.03)*
**Nonoccupational PA**				
Quintile 1	482	1.00 (0.90, 1.11)	1207	1.00 (0.94, 1.07)
Quintile 2	434	1.07 (0.97, 1.18)	934	0.99 (0.93, 1.06)
Quintile 3	385	1.02 (0.92, 1.12)	845	0.97 (0.91, 1.04)
Quintile 4	360	0.99 (0.89, 1.11)	760	0.97 (0.91, 1.05)
Quintile 5	358	1.07 (0.96, 1.20)	628	0.97 (0.89, 1.06)
per 4 units		*0.98 (0.90, 1.07)*		*0.95 (0.89, 1.01)*

Models were stratified by sex and region, and adjusted for age at baseline, education, household income, smoking, alcohol, self-rated health, diabetes, cardiovascular disease, respiratory disease, rheumatoid arthritis, and sedentary leisure time.

Supplementary Table 10. Percent change in the association of total PA with hepatobiliary diseases with additional adjustment for adiposity and diabetes

Outcome	Model	HR (95% CI)	% change		
		Overall	Overall	Male	Female
**Chronic liver disease**	Basic	0.77 (0.68, 0.87)			
	+ Waist circumference	0.83 (0.74, 0.94)	37.4 (18.6, 89.8)	47.1 (26.5, 61.3)	21.5 (10.9, 41.2)
	+ Diabetes	0.78 (0.69, 0.88)	7.2 (2.5, 20.6)	8.2 (3.9, 12.1)	2.2 (0.1, 5.5)
	+ Waist circumference, diabetes	0.84 (0.74, 0.95)	38.4 (20.4, 85.9)	51.6 (15.4, 86.4)	20.0 (10.8, 42.6)
**Liver cancer**	Basic	0.76 (0.63, 0.91)			
	+ Waist circumference	0.78 (0.65, 0.93)	8.3 (2.5, 32.5)	12.6 (1.7, 46.8)	5.5 (-5.8, 7.9)
	+ Diabetes	0.78 (0.65, 0.93)	9.4 (2.8, 26.9)	1.9 (1.4, 23.2)	5.2 (-10.8, 12.3)
	+ Waist circumference, diabetes	0.79 (0.66, 0.95)	14.6 (5.5, 50.2)	14.9 (3.6, 50.6)	28.6 (-11.4, 26.0)
**Gallstone disease**	Basic	0.86 (0.81, 0.90)			
	+ Waist circumference	0.92 (0.87, 0.97)	50.2 (29.3, 80.8)	44.9 (23.8, 93.5)	57.6 (25.6, 127.2)
	+ Diabetes	0.86 (0.82, 0.91)	5.0 (2.2, 10.3)	3.4 (0.6, 10.1)	6.1 (2.2, 16.0)
	+ Waist circumference, diabetes	0.92 (0.88, 0.97)	48.7 (29.5, 95.1)	40.0 (23.4, 100.8)	27.0 (10.2, 112.8)
**GBTC**	Basic	0.54 (0.40, 0.73)			
	+ Waist circumference	0.56 (0.41, 0.75)	4.9 (0.6, 15.3)	3.6 (0.2, 10.5)	12.6 (-66.4, 47.0)
	+ Diabetes	0.55 (0.40, 0.74)	0.5 (-1.4, 3.9)	0.8 (-1.0, 3.3)	2.8 (-19.1, 47.6)
	+ Waist circumference, diabetes	0.56 (0.41, 0.75)	4.6 (0.1, 13.1)	4.3 (0.7, 10.8)	4.4 (-72.0, 68.7)

Abbreviation: CLD, chronic liver disease; GBTC, gallbladder and biliary tract cancer; WC, waist circumference.
Models were stratified by sex and region, and adjusted for age at baseline, education, household income, smoking, alcohol, self-rated health, diabetes, cardiovascular disease, respiratory disease, rheumatoid arthritis, and sedentary leisure time. For CLD and liver cancer, the first 5 years of follow-up were excluded.

Pang Y, *et al. Br J Sports Med* 2021; 55:1024–1033. doi: 10.1136/bjsports-2020-102174

Supplementary Table 11. Associations of occupational PA with risk of hepatobiliary diseases by birth cohort

	Chronic liver disease		Liver cancer		Gallstone disease		GBTC	
	**No. cases**	**HR (95% CI)**	**No. cases**	**HR (95% CI)**	**No. cases**	**HR (95% CI)**	**No. cases**	**HR (95% CI)**
**Born before 1955**								
Quintile 1	952	1.00 (0.91, 1.10)	786	1.00 (0.89, 1.12)	1956	1.00 (0.94, 1.06)	205	1.00 (0.82, 1.22)
Quintile 2	448	0.99 (0.90, 1.10)	278	0.89 (0.78, 1.01)	935	0.97 (0.91, 1.03)	70	0.92 (0.72, 1.17)
Quintile 3	332	0.89 (0.80, 0.99)	191	0.87 (0.76, 1.01)	778	0.99 (0.93, 1.05)	59	1.02 (0.80, 1.29)
Quintile 4	319	0.92 (0.82, 1.03)	196	0.96 (0.83, 1.11)	732	0.93 (0.87, 0.99)	52	0.94 (0.73, 1.21)
Quintile 5	214	0.78 (0.68, 0.91)	146	0.79 (0.66, 0.94)	527	0.86 (0.79, 0.93)	28	0.59 (0.42, 0.84)
per 4 units		0.96 (0.93, 0.99)		0.97 (0.94, 1.01)		0.97 (0.95, 0.99)		0.90 (0.84, 0.97)
**Born after 1955**								
Quintile 1	207	1.00 (0.85, 1.18)	55	1.00 (0.73, 1.36)	470	1.00 (0.90, 1.11)	14	1.00 (0.54, 1.86)
Quintile 2	273	0.88 (0.77, 1.01)	81	1.11 (0.86, 1.42)	577	0.84 (0.77, 0.92)	14	0.94 (0.50, 1.74)
Quintile 3	360	0.72 (0.65, 0.81)	94	1.02 (0.82, 1.26)	754	0.78 (0.73, 0.84)	20	0.96 (0.61, 1.50)
Quintile 4	362	0.75 (0.67, 0.83)	108	1.13 (0.94, 1.37)	829	0.77 (0.72, 0.83)	24	1.07 (0.71, 1.61)
Quintile 5	332	0.62 (0.55, 0.70)	84	0.84 (0.67, 1.06)	1015	0.84 (0.78, 0.90)	24	0.89 (0.56, 1.39)
per 4 units		0.92 (0.90, 0.95)		0.95 (0.90, 1.01)		1.00 (0.98, 1.01)		0.97 (0.87, 1.09)
*p-value for heterogeneity**		*0.11*		*0.62*		*0.06*		*0.28*

Models were stratified by sex and region, and adjusted for age at baseline, education, household income, smoking, alcohol, self-rated health, and sedentary leisure time. Time since birth was used as the underlying time scale with delayed entry at age at baseline.

* *P*-value for heterogeneity between participants born before and after 1955.

Supplementary Table 12. E-values for total PA and hepatobiliary diseases

	RR (high vs low)	E-value
**Total PA**		
Chronic liver disease	0.74 (0.68, 0.81)	2.04
Liver cancer	0.82 (0.72, 0.94)	1.74
Gallstone disease	0.85 (0.81, 0.90)	1.63
GBTC	0.56 (0.42, 0.75)	2.97
**Total PA**		
Hospitalised NAFLD	0.62 (0.53, 0.72)	2.61
Viral hepatitis	0.73 (0.62, 0.87)	2.08
Cirrhosis	0.76 (0.66, 0.88)	1.96
Liver cancer	0.81 (0.71, 0.93)	1.77
Gallstone disease	0.86 (0.81, 0.90)	1.60
Gallbladder cancer	0.51 (0.32, 0.80)	3.33
Biliary tract cancer	0.55 (0.38, 0.78)	3.04

E-value = RR + sqrt{RR x (RR - 1)}. For factors that show inverse associations with cancer outcomes, E values are calculated using 1/RR instead of RR.

The E-values are the minimum strength of association that an unmeasured confounder need to have with the exposure and the outcome on the HR scale to fully account for an observed exposure-outcome association, above and beyond the measured covariates. The RR shows HR comparing top vs bottom quintiles of total PA.

Pang Y, *et al. Br J Sports Med* 2021; 55:1024–1033. doi: 10.1136/bjsports-2020-102174

Supplementary Table 13. Associations of total PA with hospitalised nonalcoholic fatty liver disease

Outcome	No. participants	No. cases	HR (95% CI)
Nonalcoholic fatty liver disease			
All participants			
Excluding baseline chronic liver disease	460,937	1367	0.80 (0.71, 0.91)
Participants with biochemistry*			
Excluding baseline chronic liver disease	17,478	51	0.66 (0.35, 1.24)
Excluding baseline nonalcoholic liver disease and elevated alanine aminotransferase	14,431	36	0.66 (0.32, 1.38)

* Clinical biochemistry data were available at baseline in a nested case-control study of 17,478 participants, after excluding participants with baseline cancer, gallbladder disease, hepatitis, or cirrhosis. Elevated alanine aminotransferase was defined as ≥33 U/L in men and ≥25 U/L in women.
Models were stratified by stratified by sex and region, and adjusted for age at baseline, education, household income, smoking, alcohol, self-rated health, diabetes, cardiovascular disease, respiratory disease, rheumatoid arthritis, and sedentary leisure time. Total physical activity was categorised as below and above the median (17 MET-h/day).

Supplementary Figure 1. Literature search for the meta-analysis

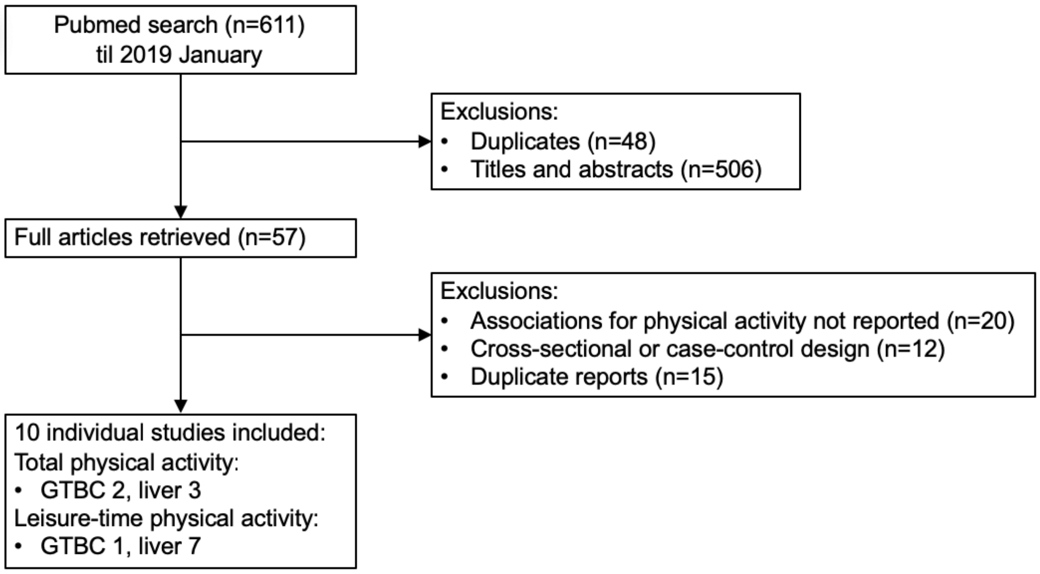

A flow chart to show selection of studies for the meta-analysis. Keywords: (liver OR gallbladder OR gallstone) AND (physical activity) AND (risk).

Pang Y, *et al. Br J Sports Med* 2021; 55:1024–1033. doi: 10.1136/bjsports-2020-102174

Supplementary Figure 2. Associations of total PA with risk of hepatobiliary cancers and diseases

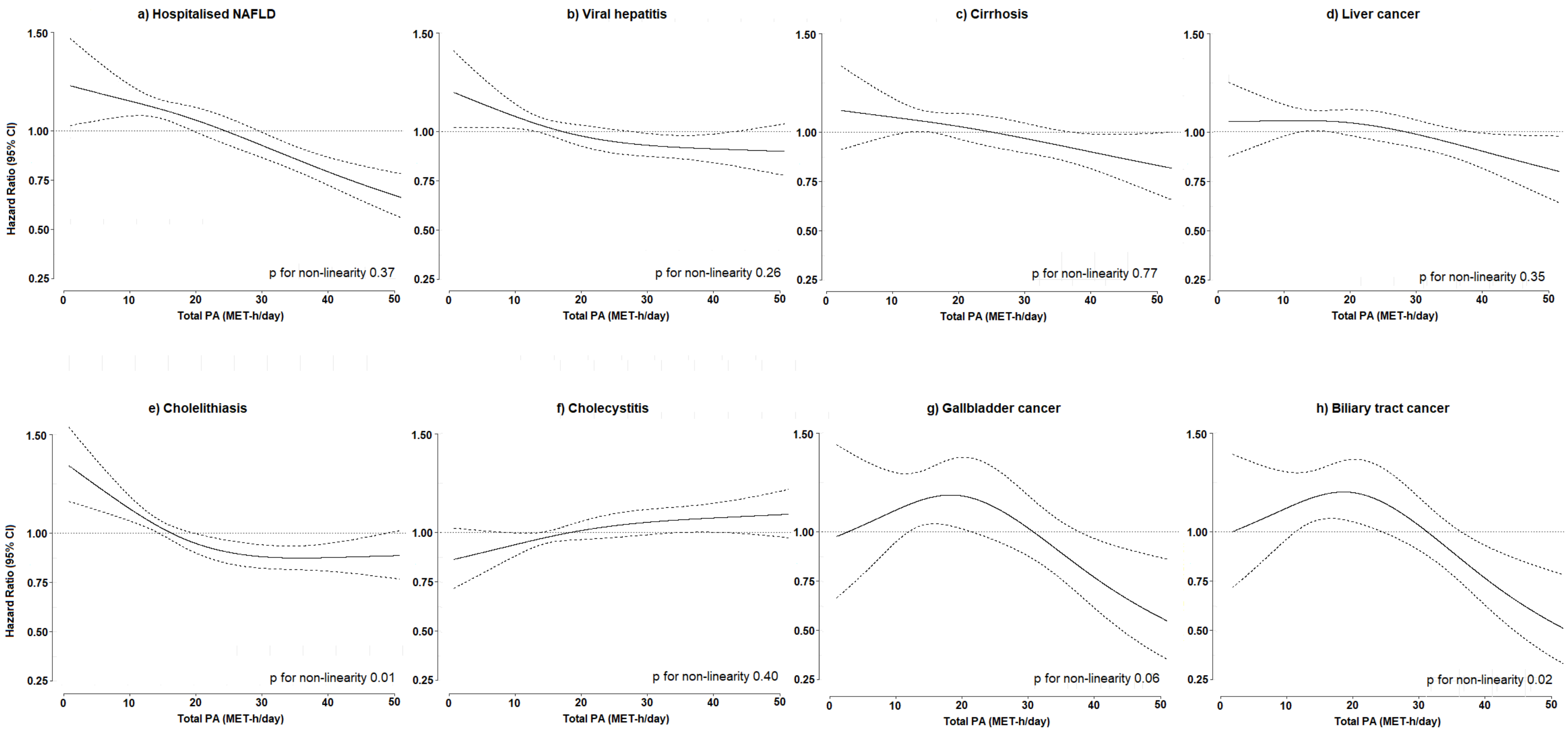

Restricted cubic splines were calculated using three fixed knots at the 10%, 50%, and 90% quantiles. Models were stratified by sex and region, and adjusted for age at baseline, education, household income, smoking, alcohol, self-rated health, and sedentary leisure time. Time since birth was used as the underlying time scale with delayed entry at age at baseline.

Supplementary Figure 3. Associations of occupational and nonoccupational PA with risk of liver diseases and cancer

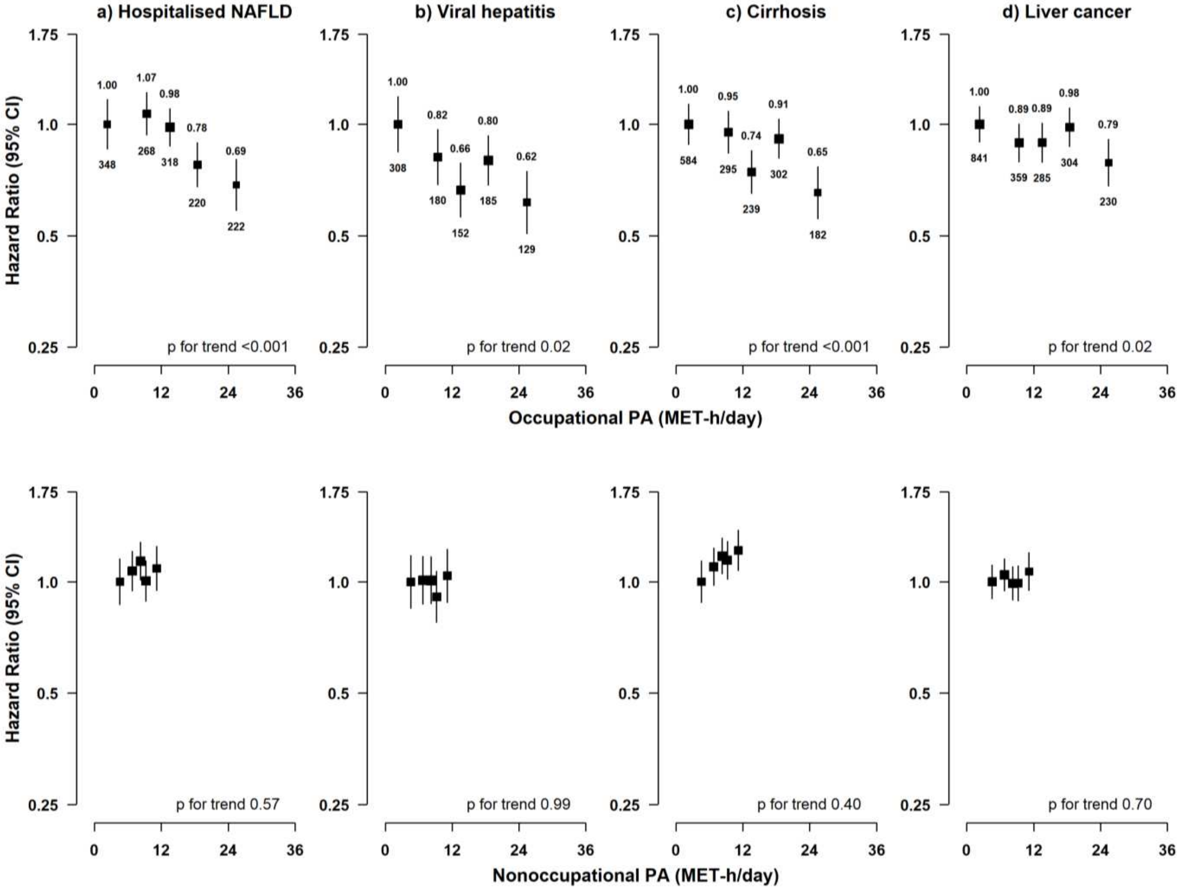

Models were stratified by sex and region, and adjusted for age at baseline, education, household income, smoking, alcohol, self-rated health, and sedentary leisure time. Time since birth was used as the underlying time scale with delayed entry at age at baseline. HRs are plotted against the mean level in each category of physical activity. Log-scale is used for the y-axis. The squares represent HRs and the vertical lines represent 95% CIs. The area of the squares is inversely proportional to the variance of the log HRs. The numbers above the vertical lines are point estimates for HRs, and the numbers below the lines are numbers of events.

Supplementary Figure 4. Associations of occupational and nonoccupational PA with risk of gallbladder diseases and GBTC

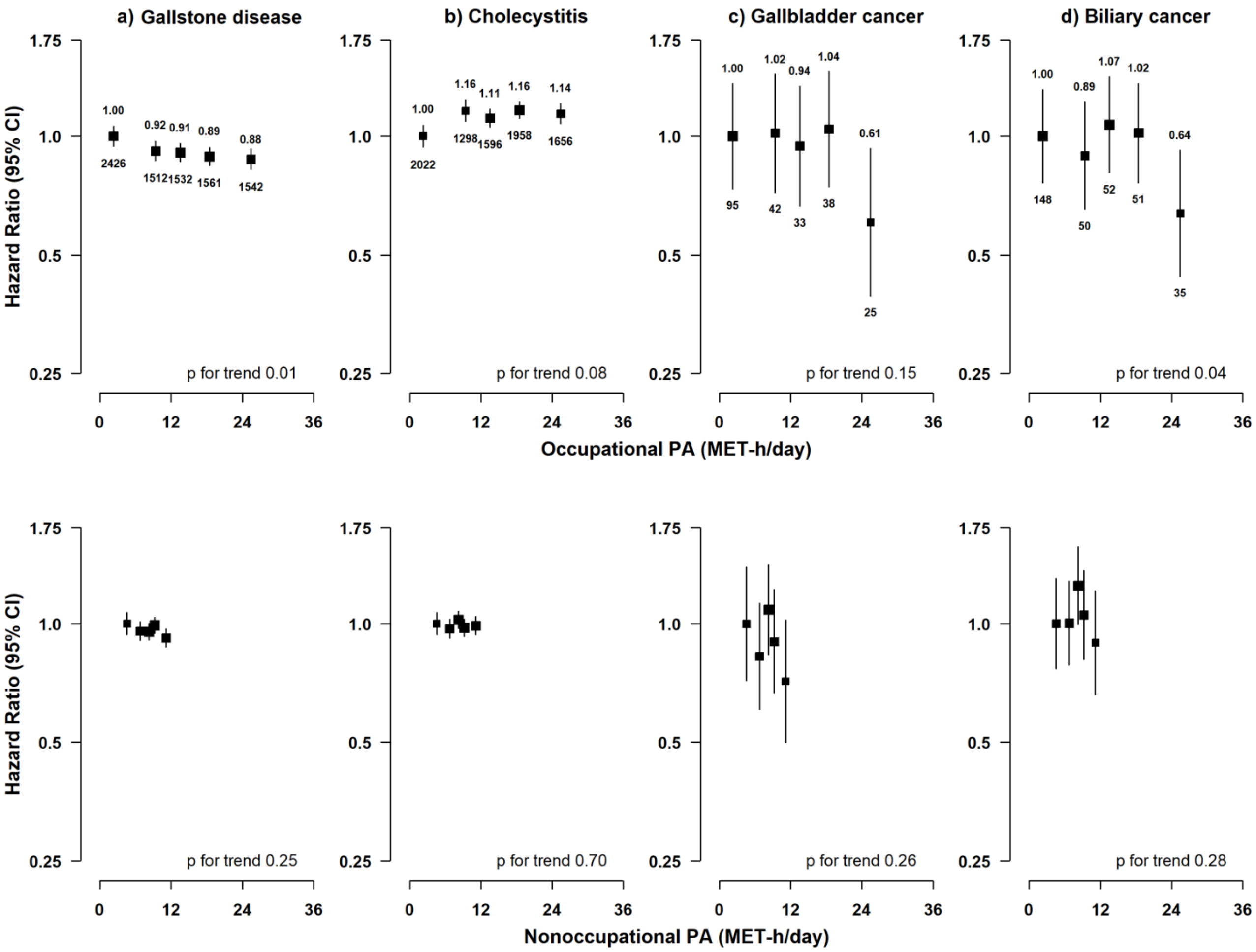

Convention as is Supplementary Figure 3.

Supplementary Figure 5. Associations of domain-specific PA with risk of hepatobiliary disease by region

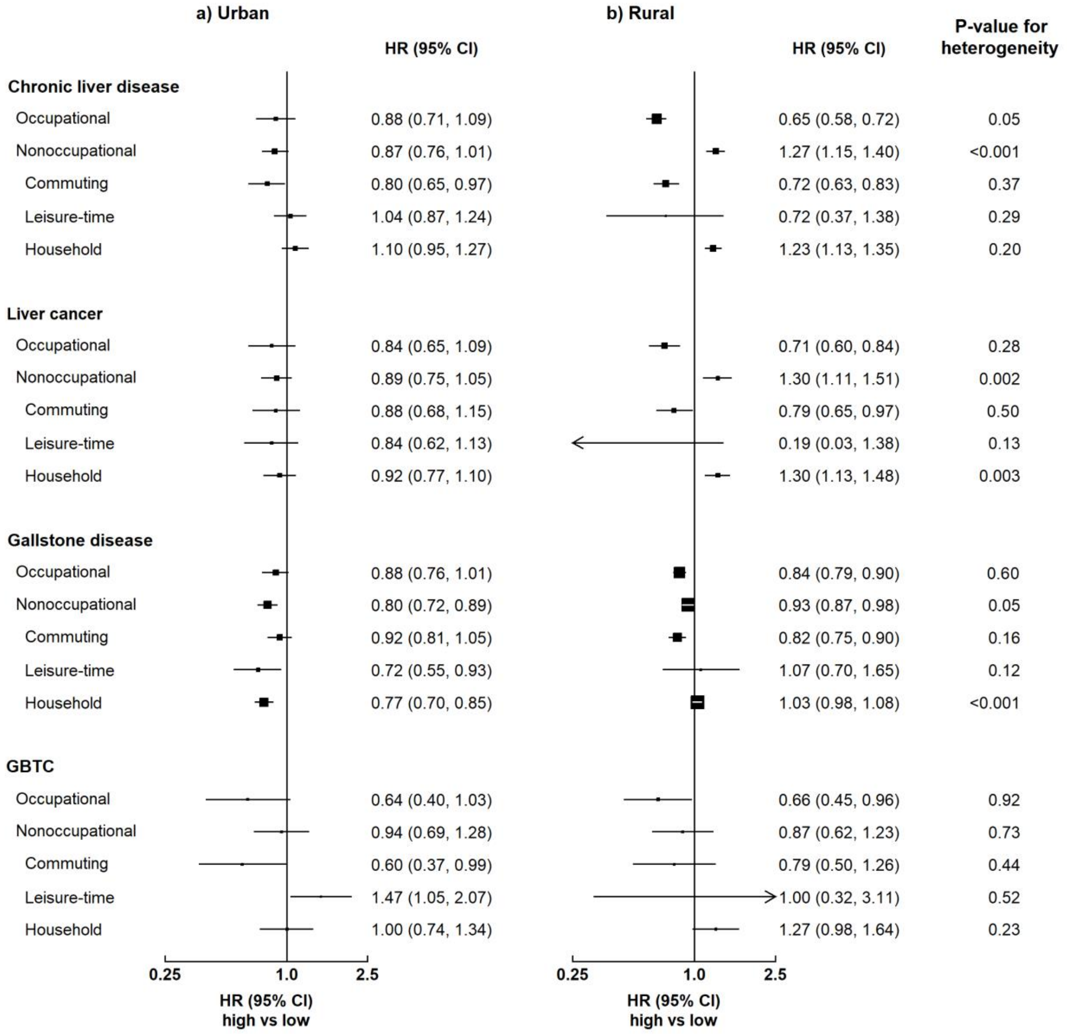

Models were stratified by sex and region, and adjusted for age at baseline, education, household income, smoking, alcohol, self-rated health, diabetes, cardiovascular disease, respiratory disease, rheumatoid arthritis, and sedentary leisure time. The amount of domain-specific physical activity was categorised by splitting at quintiles. To ensure enough participants in each category of physical activity, we estimated HRs comparing the highest quintile with the lower four quintiles of total physical activity for these subgroup analyses. For leisure-time physical activity, the HR was comparing ≥7.5 MET-h/day to none.

Pang Y, *et al. Br J Sports Med* 2021; 55:1024–1033. doi: 10.1136/bjsports-2020-102174

Supplementary Figure 6. Associations of domain-specific PA with risk of hepatobiliary disease by sex

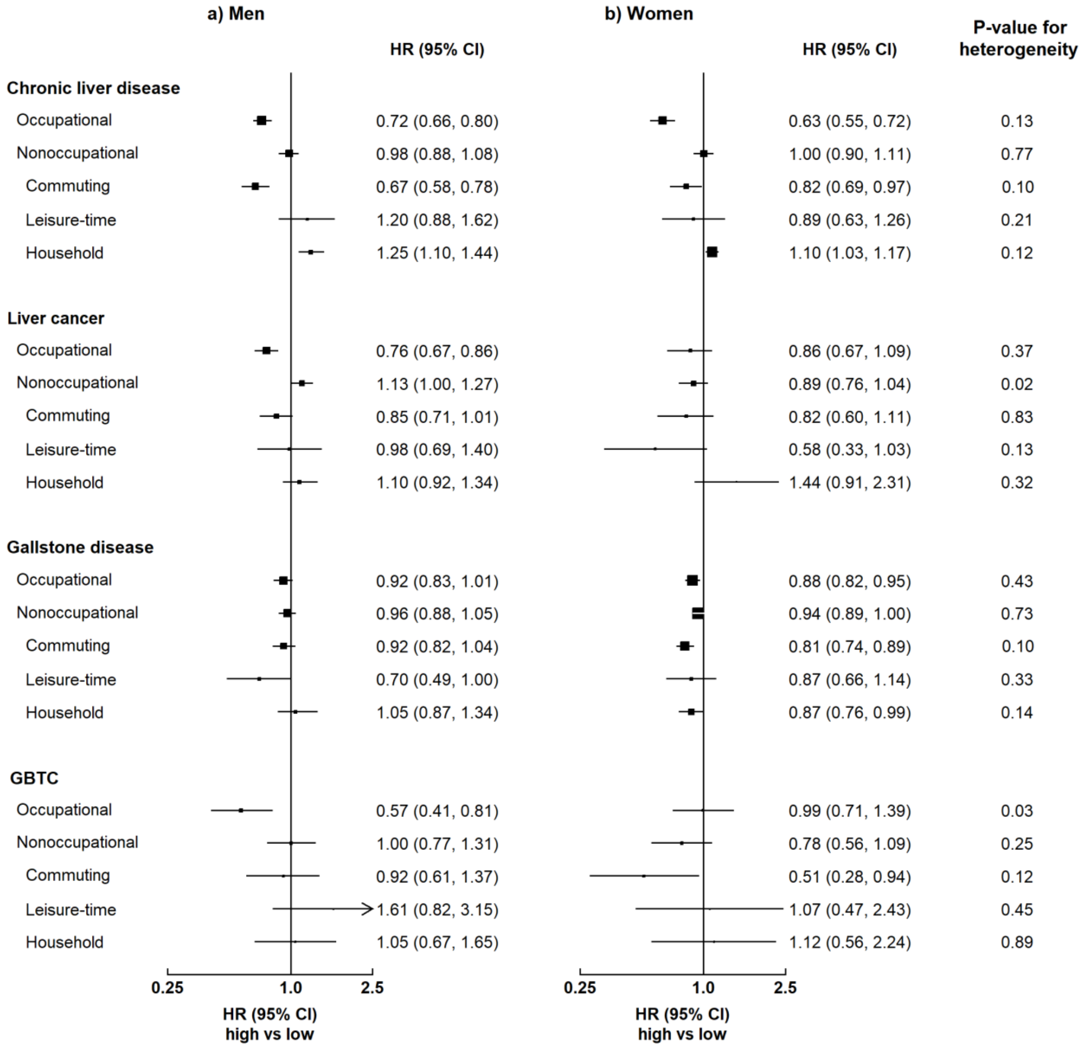

Models were stratified by region, and adjusted for age at baseline, education, household income, smoking, alcohol, self-rated health, diabetes, cardiovascular disease, respiratory disease, rheumatoid arthritis, and sedentary leisure time. The amount of domain-specific physical activity was categorised by splitting at quintiles. To ensure enough participants in each category of physical activity, we estimated HRs comparing the highest quintile with the lower four quintiles of total physical activity for these subgroup analyses. For leisure-time physical activity, the HR was comparing ≥7.5 MET-h/day to none.

Supplementary Figure 7. Associations of total PA with risk of chronic liver disease and liver cancer by participant characteristic

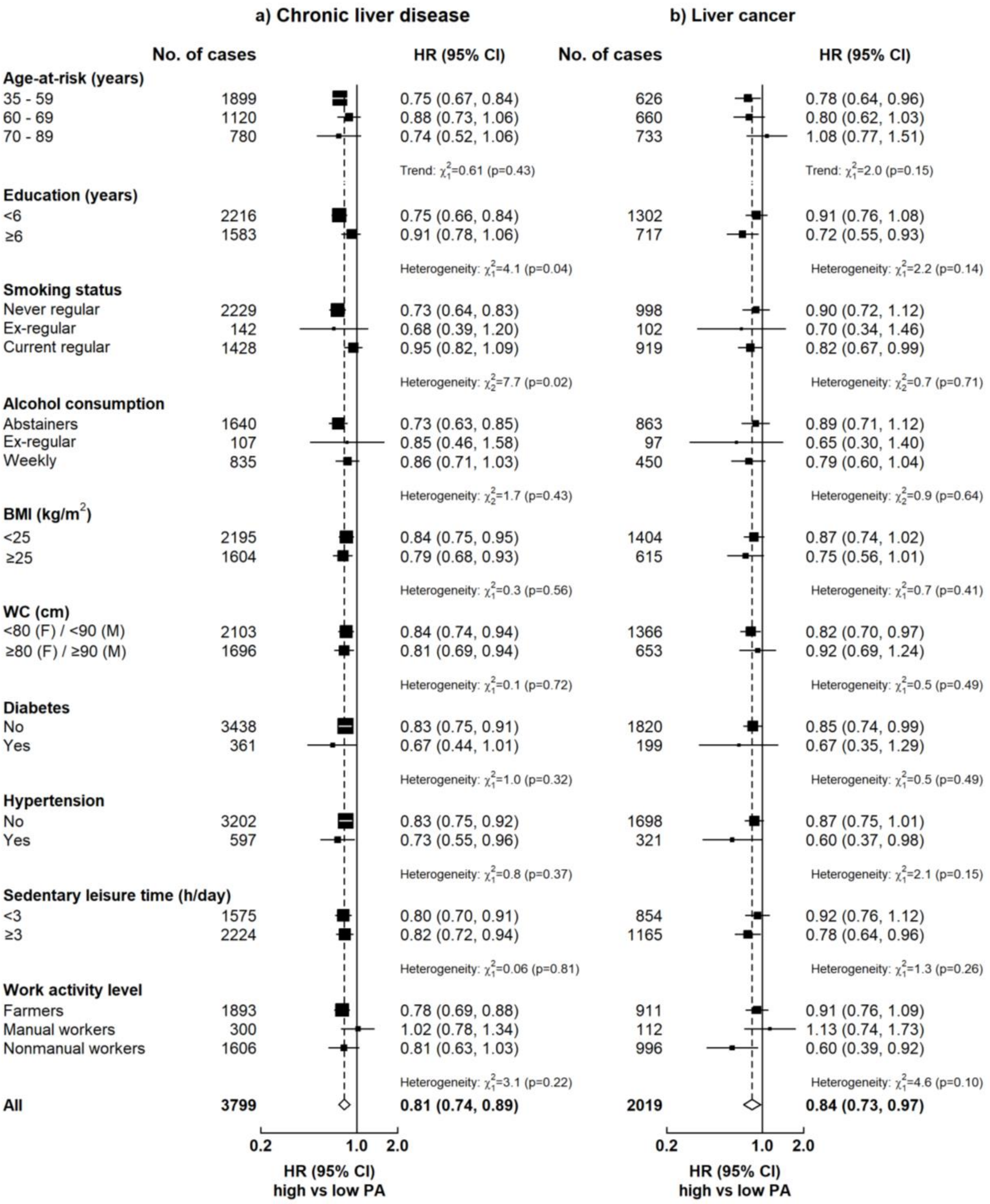

Models were stratified by sex and region, and adjusted for age at baseline, education, household income, smoking, alcohol, self-rated health, diabetes, cardiovascular disease, respiratory disease, rheumatoid arthritis, and sedentary leisure time, where appropriate. To ensure enough participants in each category of physical activity, we estimated HRs comparing the highest quintile with the lower four quintiles of total physical activity for these subgroup analyses.

Supplementary Figure 8. Associations of total PA with risk of gallstone disease and GBTC by participant characteristic

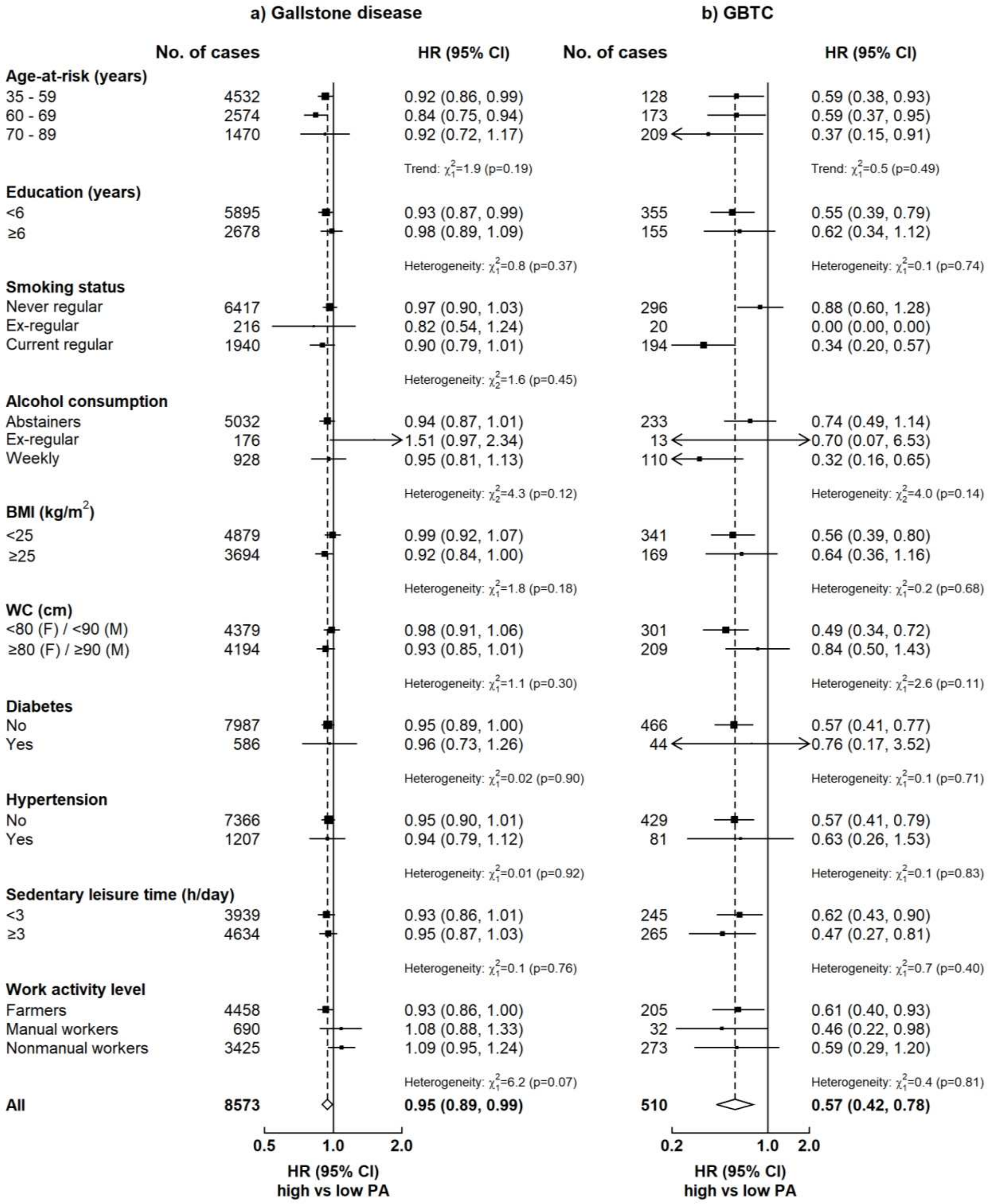

Convention as is Supplementary Figure 7.

Pang Y, *et al. Br J Sports Med* 2021; 55:1024–1033. doi: 10.1136/bjsports-2020-102174

Supplementary Figure 9. Associations of total PA with risk of hepatobiliary diseases with different exclusions

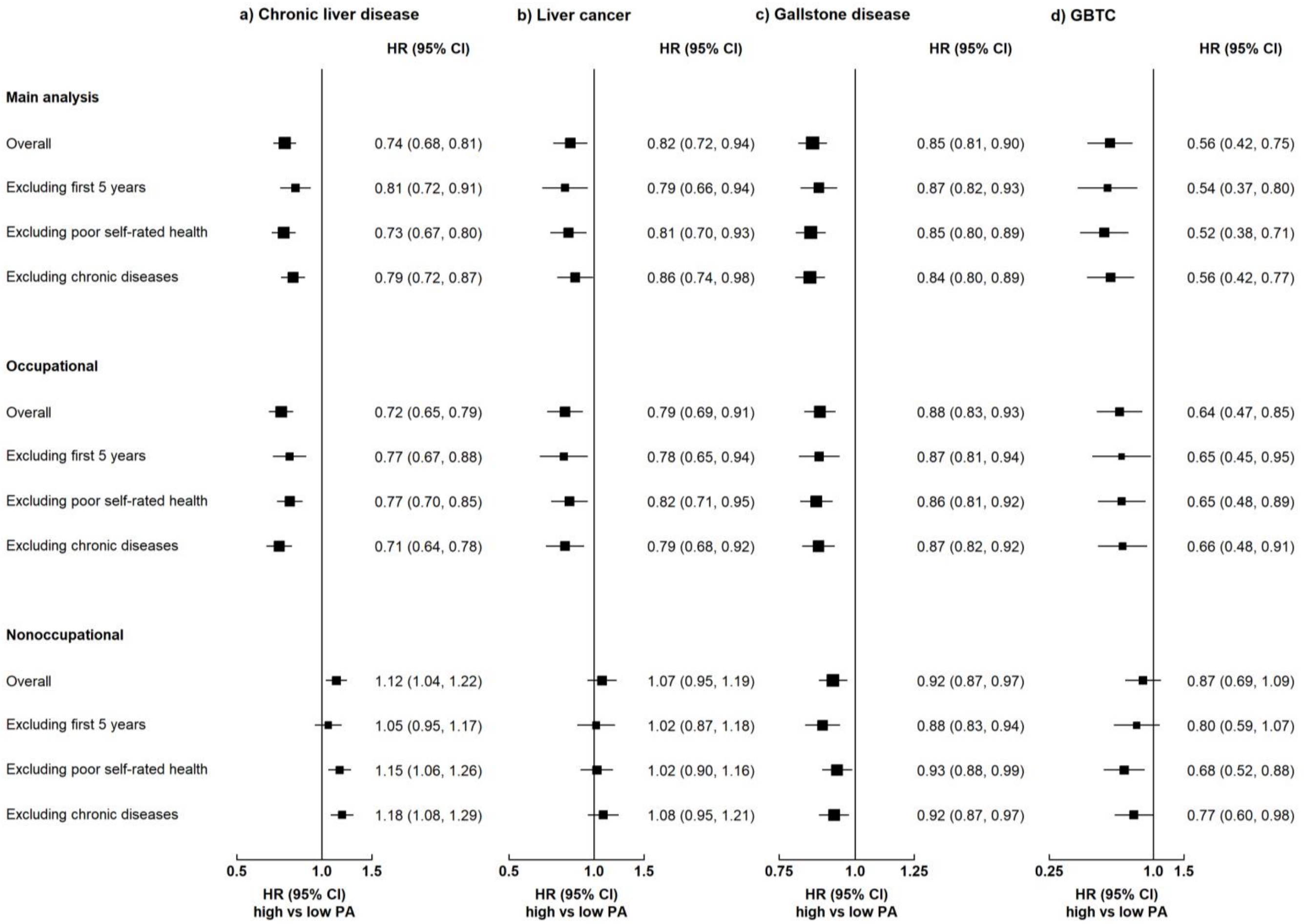

Convention as is Supplementary Figure 7**.**

Supplementary Figure 10. Associations of domain-specific PA with risk of liver cancer and accidental death

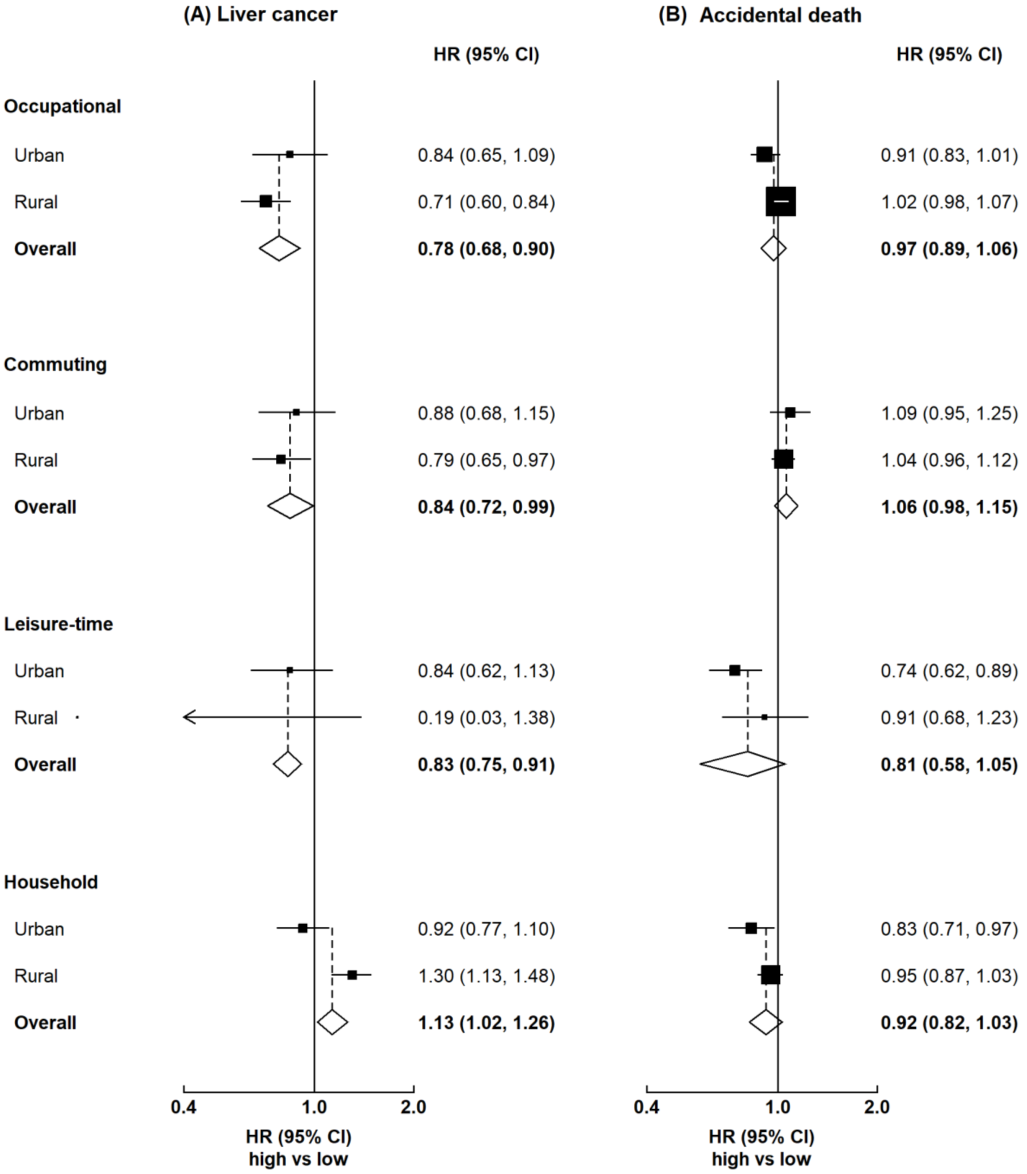

Convention as is Supplementary Figure 5.

Pang Y, *et al. Br J Sports Med* 2021; 55:1024–1033. doi: 10.1136/bjsports-2020-102174

Supplementary Figure 11. Meta-analysis of prospective studies on PA and hepatobiliary diseases

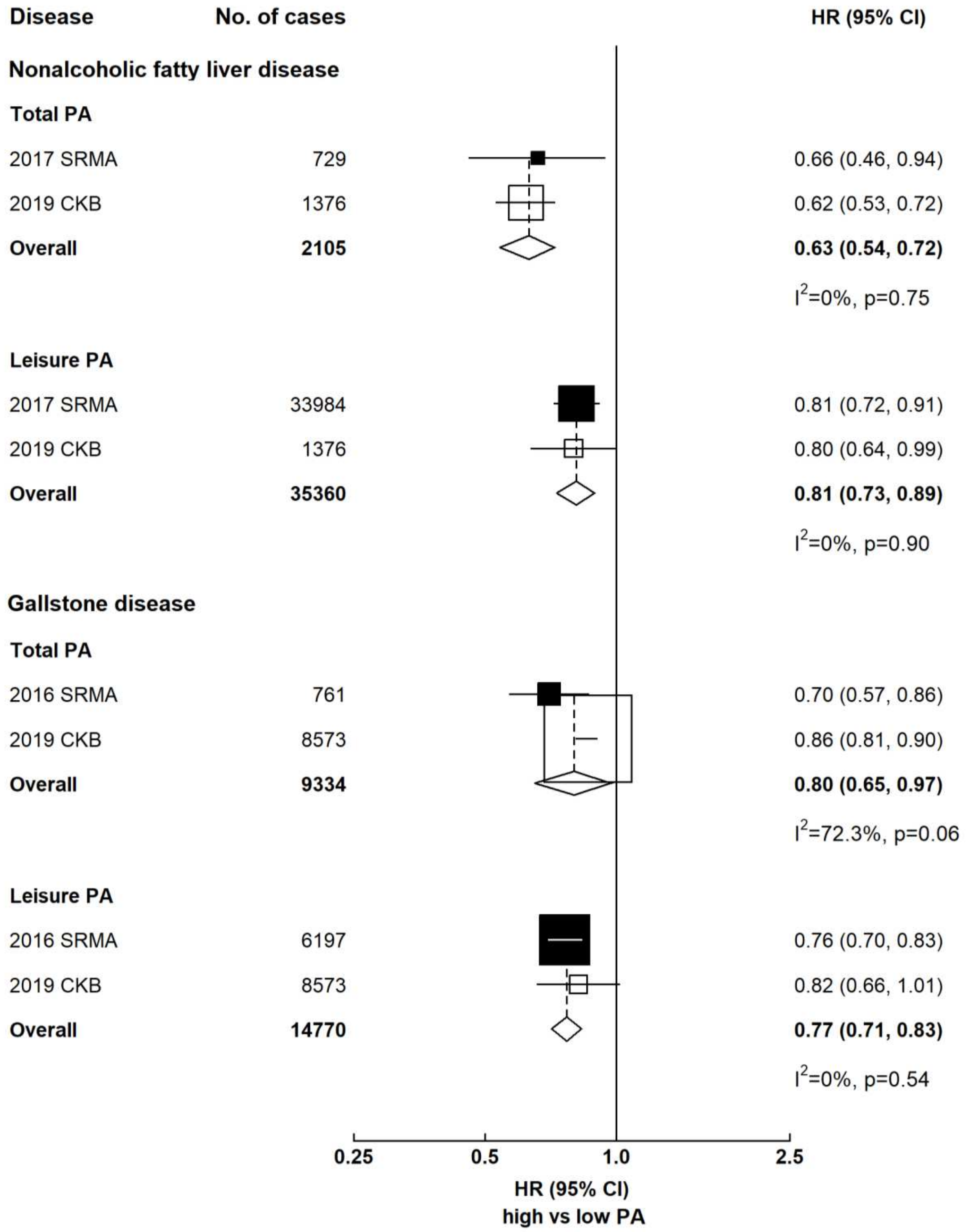

Boxes represent the hazard ratios (HRs) associated with hepatobiliary cancer comparing high vs low categories of physical activity, with the size of the box inversely proportional to the variance of the logHR. Open boxes represent previously published studies and the black boxes represent CKB estimates. Diamonds represent summary HRs for overall. Within categories HRs are ordered according to their year of publication. Estimates and 95% CI of the summary HRs are in bold. Abbreviation: SRMA, systematic review and meta-analysis. Reference: 2017 SRMA, PMID 28932271; 2016 SRMA, PMID 26901710.

Pang Y, *et al. Br J Sports Med* 2021; 55:1024–1033. doi: 10.1136/bjsports-2020-102174

Supplementary Figure 12. Associations of total PA with risk of nonalcoholic fatty liver disease by participant characteristic

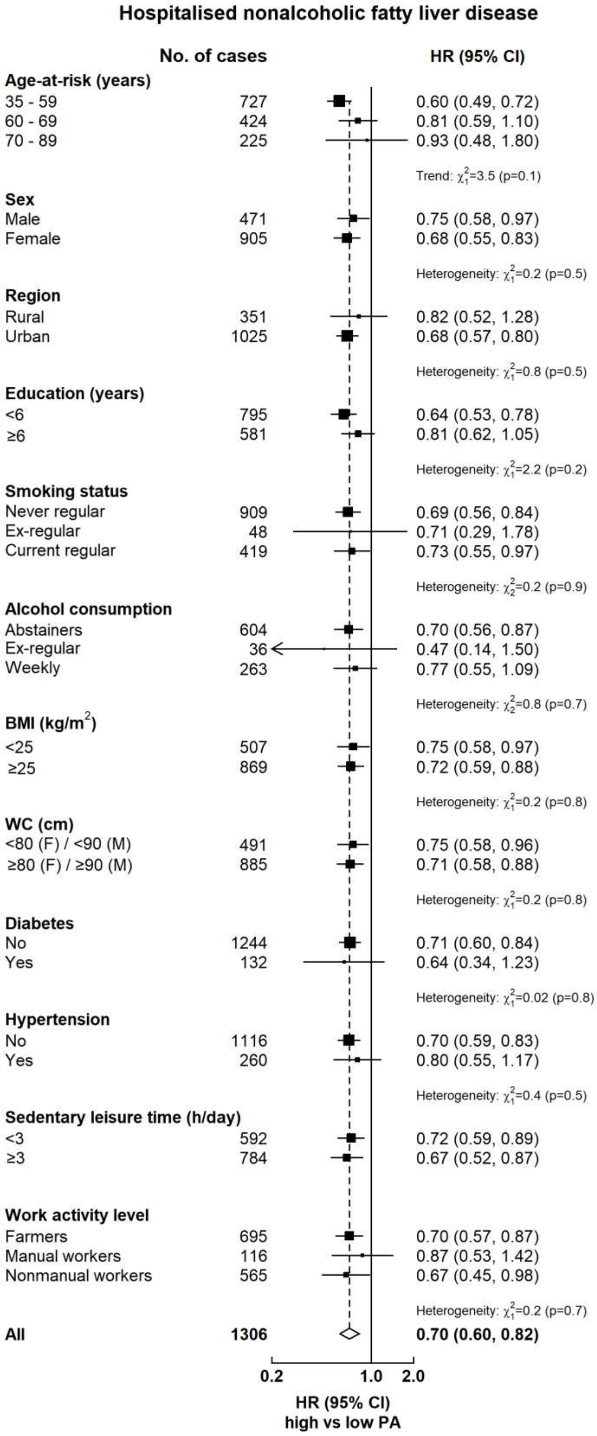

Models were stratified by sex and region, and adjusted for age at baseline, education, household income, smoking, alcohol, diabetes, cardiovascular disease, respiratory disease, rheumatoid arthritis, and sedentary leisure time, where appropriate. To ensure enough participants in each category of physical activity, we estimated HRs comparing the highest quintile with the lower four quintiles of total physical activity for these subgroup analyses.

Pang Y, *et al. Br J Sports Med* 2021; 55:1024–1033. doi: 10.1136/bjsports-2020-102174
